# A carotenoid-deficient mutant of the plant-associated microbe *Pantoea* sp. YR343 displays an altered membrane proteome

**DOI:** 10.1038/s41598-020-71672-w

**Published:** 2020-09-11

**Authors:** Sushmitha Vijaya Kumar, Paul E. Abraham, Gregory B. Hurst, Karuna Chourey, Amber N. Bible, Robert L. Hettich, Mitchel J. Doktycz, Jennifer L. Morrell-Falvey

**Affiliations:** 1 UT-ORNL Graduate School of Genome Science and Technology, University of Tennessee, Knoxville, TN USA; 2 Chemical Sciences Division, Oak Ridge National Laboratory, Oak Ridge, TN USA; 3 Department of Biochemistry and Cellular and Molecular Biology, University of Tennessee, Knoxville, TN USA; 4 Biosciences Division, Oak Ridge National Laboratory, Oak Ridge, TN USA; 5 Center for Nanophase Materials Sciences, Oak Ridge National Laboratory, Oak Ridge, TN USA

**Keywords:** Microbiology, Proteomic analysis

## Abstract

Membrane organization plays an important role in signaling, transport, and defense. In eukaryotes, the stability, organization, and function of membrane proteins are influenced by certain lipids and sterols, such as cholesterol. Bacteria lack cholesterol, but carotenoids and hopanoids are predicted to play a similar role in modulating membrane properties. We have previously shown that the loss of carotenoids in the plant-associated bacteria *Pantoea *sp. YR343 results in changes to membrane biophysical properties and leads to physiological changes, including increased sensitivity to reactive oxygen species, reduced indole-3-acetic acid secretion, reduced biofilm and pellicle formation, and reduced plant colonization. Here, using whole cell and membrane proteomics, we show that the deletion of carotenoid production in *Pantoea sp.* YR343 results in altered membrane protein distribution and abundance. Moreover, we observe significant differences in the protein composition of detergent-resistant membrane fractions from wildtype and mutant cells, consistent with the prediction that carotenoids play a role in organizing membrane microdomains. These data provide new insights into the function of carotenoids in bacterial membrane organization and identify cellular functions that are affected by the loss of carotenoids.

## Introduction

Biological membranes serve as a central scaffold for cellular machinery that regulate key physiological functions including signaling, defense, metabolism, and molecular transport^1,2^. Indeed, genes encoding membrane proteins account for 20–30% of the entire genetic complement of a bacterial cell^3,4^. These membrane proteins are important for processes such as cell motility, chemotaxis, cyclic dimeric guanosine monophosphate (c di-GMP) signaling, virulence, multidrug efflux, and outer membrane biogenesis^5–7^. Bacterial outer membrane proteins are also directly involved in bacterial acclimatization by monitoring and responding to environmental cues.

Apart from proteins and lipids, some membranes contain carotenoids. These compounds have been implicated in photoprotection, imparting coloration to plants, animals and bacteria, and used as a chromophore in photosynthesis^8–11^. In the photosynthetic apparatus of plants, algae, and bacteria, carotenoids are found in the light-harvesting pigment-protein complexes^12^. In bacterial membranes, carotenoids have been shown to reinforce membranes and modulate membrane thickness and fluidity^13–17^. These properties are essential for many key molecular processes, such as signal transduction, that involve the movement of proteins in the membrane. Therefore, changes in the structure and dynamics of a membrane due to carotenoids can further affect cellular events occurring at the membrane.

The existence of membrane microdomains has traditionally been associated with eukaryotic membranes, but recent studies have shown that some bacteria, such as *Bacillus subtilis*, can form functional membrane microdomains^18^. The formation of these microdomains in prokaryotes is thought to involve sterol analogs, such as hopanoids and carotenoids^18,19^. Eukaryotic membrane microdomains, sometimes called lipid rafts, are characterized by the presence of cholesterol and flotillin^18–20^. The activity of flotillin in eukaryotes is critical for the functioning of lipid raft-associated cellular processes, such as membrane trafficking and cell polarization^21–23^. Homologs of flotillin have also been identified in prokaryotes^24–26^ and these prokaryotic flotillins also appear to organize the membrane into domains, enabling protein interactions and oligomerization^19,25,27^. This lateral membrane organization and sub-compartmentalization is critical for efficient membrane function^28^.

We have shown previously that the loss of carotenoids in the plant-associated bacteria *Pantoea* sp. YR343 results in cells that are more susceptible to oxidative damage, but also display defects in plant root colonization, biofilm formation, and in indole-3-acetic acid secretion^29^. Lipid profiling of this carotenoid-deficient mutant (generated by deleting the *crtB* gene encoding phytoene synthase) demonstrated that the mutant strain displays an increase in phosphatidylethanolamine and unsaturated fatty acids when compared to wildtype cells^17^. Moreover, these differences in lipid profiles also translate to differences in membrane fluidity between the wildtype and the Δ*crtB* mutant^17^. From these studies, it is evident that the loss of carotenoids from the membrane leads to changes in membrane properties and organization, thereby influencing cellular functions. In this paper, we examine the proteomic profile of Δ*crtB* mutant cells compared to wildtype to better understand the role of carotenoids in membrane organization. To this end, we compare proteomic profiles of whole cells, membrane fractions, and the distribution of membrane proteins in detergent-resistant membrane (DRM) and detergent-sensitive membrane (DSM) fractions.

## Results and discussion

### Identification and quantification of protein abundances in *Pantoea* sp. YR343 and Δ*crtB* whole cells, membrane pellet, detergent resistant membranes (DRM) and detergent sensitive membrane (DSM) samples

**Figure 1:**
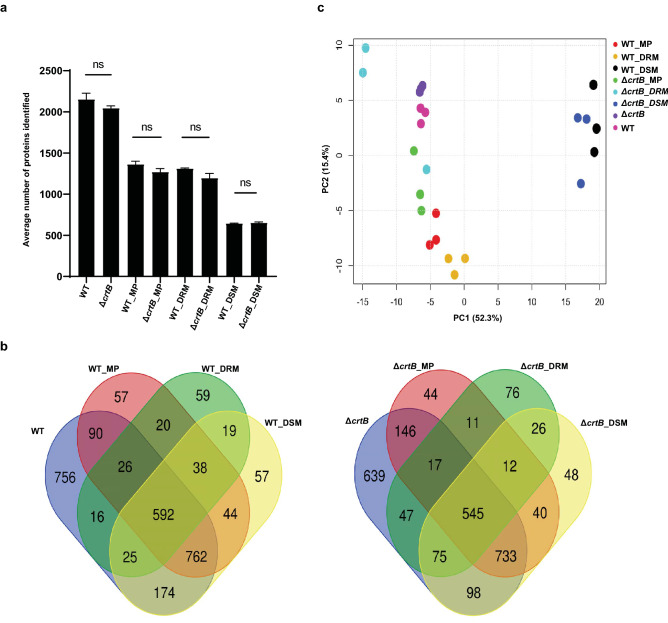
Identification and analysis of the proteins identified in whole cell, membrane pellet, DRM and DSM fractions of *Pantoea* sp. YR343 and Δ*crtB* mutant cells. (**a**) Histogram representing the number of proteins (averaged from 3 biological replicates) identified from whole cell, membrane fraction (MP), detergent resistant membrane fraction (DRM) and detergent sensitive membrane fractions (DSM) for wild type and the Δ*crtB* mutant using proteomics. Statistical significance was calculated by One-way ANOVA. *ns* non-significant. (**b**) Venn diagrams comparing the number of identified proteins in common between different samples (whole cell, MP, DRM and DSM). Each ellipse from WT or the mutant represent whole cell, MP, DRM or DSM samples, with the number of common proteins between samples shown in the overlapping regions. The number in the non-overlapping region represents unique proteins for each sample. (**c**) Comparison of samples using principle component analysis (PCA) based on normalized abundance. The plot illustrates discrete grouping of biological replicates with a major variance observed in PC1 for the DSM samples and in PC2 across the remaining factors.

Because we observed changes in the membrane lipid profiles and membrane fluidity in *Pantoea* sp. YR343 cells lacking carotenoids^17^, we wanted to determine how the loss of carotenoids affected the membrane proteome. To this end, whole cell, membrane pellet (MP), DRM, and DSM samples from *Pantoea* sp. YR343 and Δ*crtB* strains harvested during stationary phase were used for proteome characterization. Equal amounts of protein from each fraction were analyzed by proteomics, although we measured reduced protein concentrations from the mutant membrane, DRM, and DSM fractions (see Methods). Overall, an average of 2,153 and 2046 proteins were identified for wildtype and Δ*crtB* whole cells, with an average proteome coverage of 44% and 42% respectively. For the membrane fractions investigated, 1,363 (28%) and 1,270 (26%) proteins were identified for wildtype and Δ*crtB* MP samples, 1,311 (27%) and 1,196 (24%) proteins were identified for the wildtype and Δ*crtB* DRM samples and 645 (13%) and 653 (13%) proteins were identified for wildtype and Δ*crtB* DSM samples (Fig. 1a). A Venn diagram comparing the proteins identified in four different conditions is shown in Fig. 1b, illustrating the intersections between proteomes. A total of 592 and 545 proteins were found in all four wildtype and Δ*crtB* mutant samples, respectively.

Principle component analysis (PCA) (Fig. 1c) of the proteome data indicated distinct groupings for proteins isolated from the whole cell, membrane pellet, and DSM fractions in each of the wildtype and Δ*crtB* biological replicates (n = 3), indicating reproducibility between replicates as well as similarity between the two strains (wildtype and Δ*crtB* mutant). In contrast, a scattered distribution was observed for the wildtype and *ΔcrtB* DRM samples (Fig. 1c). Major variance was observed in PC1 (discrete grouping of samples) with DSM samples and in PC2 (major variance) across the remaining factors. Sterols are vital components in the formation of membrane microdomains^19^ and proteins that localize to lipid rafts or microdomains are typically found in the detergent-resistant membrane fractions^18,30^. Thus, the absence of carotenoids may affect the formation, stability, or recruitment of proteins to microdomains, resulting in the observed differences between the wildtype and Δ*crtB* mutant DRM fractions. Proper functioning of proteins is regulated by membrane bilayer properties such as lipid curvature, bilayer thickness, and elastic properties provided by sterols^31^. Molecular dynamics simulations of the carotenoid zeaxanthin with 1,2-dimyristoyl-sn-glycero-3-phosphocholine (DMPC) bilayers have shown that carotenoids influence the physical properties of bilayers^32^. Unlike cholesterol which can only span one leaflet of the lipid bilayer, a C40 carotenoid can span both leaflets^14^. A recent study has shown that zeaxanthin can trigger the bilayer to become thinner through the process of interdigitation or compression^16^. It has been well established that bilayer thickness, which is modulated by phospholipid chain composition and sterol content, is a critical factor for the proper insertion and function of membrane proteins^33,34^. Thus, the differences in protein composition and abundance detected in the Δ*crtB* mutant could be due to changes in membrane thickness as a result of the loss of carotenoids.

**Figure 2:**
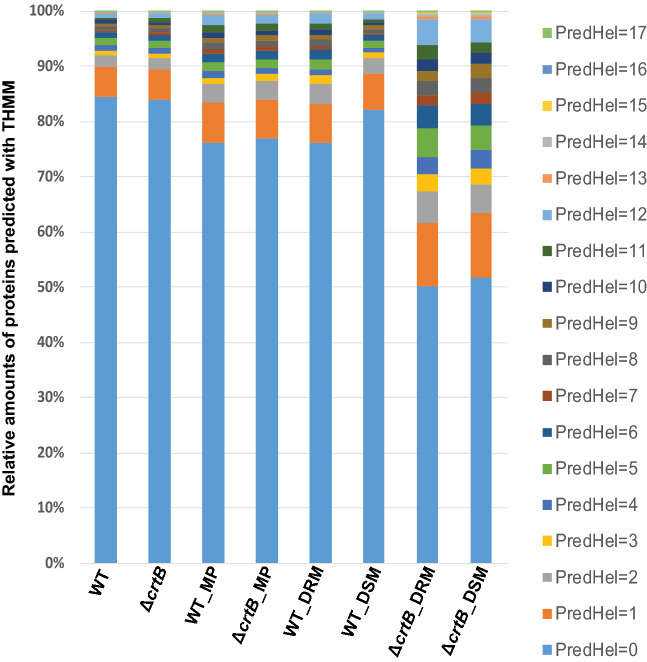
Histogram representing the percentage of proteins with predicted transmembrane helix domains (TMHMM) for each sample. Proteins with predicted transmembrane helices were identified using TMHMM software. The membrane fraction samples contain higher relative amounts of proteins with predicted TMHMM domains, with the DSM fraction having the largest enrichment.

To test for the enrichment of membrane proteins in each fraction, we used TMHMM software, a membrane protein topology prediction method, in order to identify proteins containing at least one transmembrane helix, with a maximum number of transmembrane helices detected at 17. Approximately 24% of the proteome of *Pantoea* sp. YR343 consists of proteins with at least one transmembrane helix. As shown in Fig. 2, the largest enrichment of proteins with transmembrane helices for this study was found in the wildtype and *ΔcrtB*-DSM samples. In general, the proportion of membrane proteins to other cellular proteins is low and the limitations of solubility and separation may limit their detection and identification^35^. While the proportion of proteins with transmembrane helices was similar between the wildtype and mutant in the whole cell, membrane fraction, and DSM fractions, we observed that the Δ*crtB* DRM fraction had fewer proteins with transmembrane helices compared to the wildtype DRM fraction (Fig. 2). This result is consistent with the idea that changes in membrane thickness or microdomain organization due to loss of carotenoids influences protein recruitment or localization, as mentioned previously. It should be noted that this analysis cannot identify membrane-associated proteins that might also be influenced by the loss of carotenoids. In addition, membrane microdomains are known to be transient structures and their identification depends on the state of cellular activity^36^. For this reason, it is possible that the observed proteome differences between wildtype and mutant cells are due to physiological differences between the cultures, even though care was taken to harvest both cultures during stationary phase.

**Figure 3:**
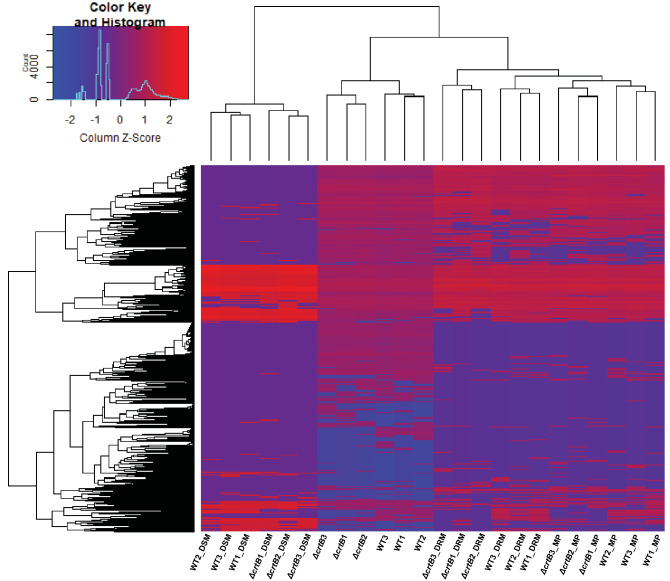
Hierarchical clustering of all proteins identified in *Pantoea* sp. YR343 wildtype and the Δ*crtB* mutant. Heatmap of protein counts in *Pantoea* sp. YR343 wildtype and the *ΔcrtB* mutant indicate fraction specific abundance of proteins and differential abundance of proteins between wildtype and the Δ*crtB* mutant. Higher protein abundance is indicated by red and lower protein abundance is indicated by blue. The heatmap was generated using gplots in Rstudio and scaled by column.

**Figure 4:**
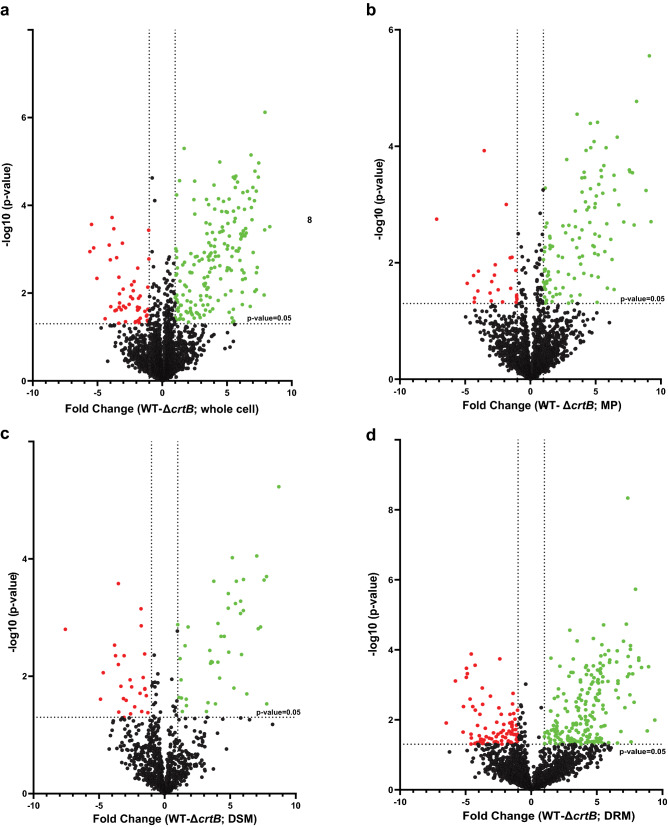
Volcano plots illustrating significantly differentially abundant proteins. The − log10 *p* value (Benjamini–Hochberg corrected) (y-axis) is plotted against the fold change (x-axis) to identify significantly different proteins between sample types. Proteins with significantly increased (green) or decreased (red) abundance are shown for (**a**) Whole cell fraction; (**b**) Membrane pellet fraction; (**c**) DSM fraction; and (**d**) DRM fraction. The dashed line represents a significance level of *p* ≤ 0.05 (Student’s t-test).

Next, hierarchical clustering was used to assess the differences in abundance of all proteins identified in the four fractions for wildtype and Δ*crtB* cells (Fig. 3). From this analysis, we found that each sample fraction (whole cell, membrane, DRM, or DSM) clustered together. A Student’s t-test (paired t-test) was performed and a *p* value cutoff of ≤ 0.05 and a fold change (FC) of ≥ 2 was used to identify proteins with relative abundances that significantly differed between the wildtype and Δ*crtB* samples. In total, 240, 134, 297 and 71 proteins were differentially abundant between wildtype and Δ*crtB* cells for the whole cell, MP, DRM and DSM fractions, respectively (Fig. 4). Out of these, 188 (in whole cell), 111 (in MP), 211 (in DRM) and 44 (in DSM), were significantly less abundant in the Δ*crtB* mutant in comparison to the wildtype, In total, 21 proteins were found to be differentially abundant across all four fractions in both wild type and Δ*crtB* cells. The observed phenotypes found in the Δ*crtB* mutant may be a result of the changes in abundance or distribution of these identified proteins.

### Cluster of orthologous groups (COG) analysis

**Figure 5:**
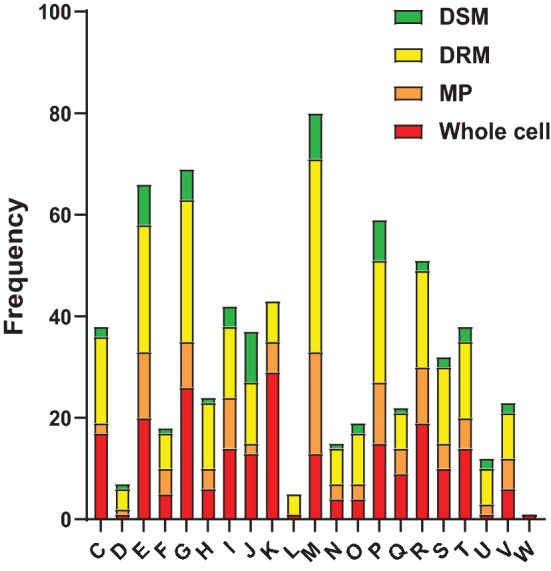
Top orthologous groups for all significant proteins in whole cell, membrane pellet, DRM and DSM samples. The functional classification of statistically significant proteins was organized according to COG assignments. The proteins in each COG category are proportioned based on the fraction in which they are differentially abundant with red indicating whole cell fraction, orange indicating membrane fraction, yellow indicating DRM fraction, and green indicating DSM fraction. The COG category labels are as follows: E-Amino acid transport and metabolism; G-Carbohydrate transport and metabolism; D-Cell cycle control, cell division, chromosome partitioning; N-Cell motility; M-Cell wall/membrane/envelope biogenesis; H-Coenzyme transport and metabolism; V-Defense mechanisms; C-Energy production and conversion; W-Extracellular structures; S-Function unknown R-General function prediction only; P-Inorganic ion transport and metabolism; U-Intracellular trafficking, secretion, and vesicular transport; I-Lipid transport and metabolism; F-Nucleotide transport and metabolism; O-Post-translational modification, protein turnover, and chaperones; L-Replication, recombination and repair; Q-Secondary metabolites biosynthesis, transport, and catabolism; T-Signal transduction mechanisms; K-Transcription; J-Translation, ribosomal structure and biogenesis.

To identify biological processes related to the differentially abundant proteins, functional classification of significant proteins was carried out using the COG database^37^. Significant proteins were grouped into 21 functional classes according to COG classification. Proteins belonging to transcription (K) and carbohydrate transport and metabolism (G) categories were abundant in whole cell samples, whereas cell wall/membrane/envelope biogenesis (M) proteins were abundant in membrane pellet, DRM, and DSM fractions (Fig. 5).

### Gene ontology (GO) enrichment analysis

To gain a deeper understanding of overall changes in protein abundance and distribution between the wildtype and Δ*crtB* mutant, functional in silico classification of proteins was achieved via GO analysis using the BLAST2GO tool^38^. All of the proteins that were differentially abundant (*p* value ≤ 0.05 and a FC ≥ 2) based on the proteomic analyses were organized by GO terms to determine which biological processes were affected by the loss of carotenoids (Table 1).

**Table 1:** Whole-genome gene ontology (GO) term annotation using Blast2GO software.

GOID	GO term	Ontology source	No. of genes
**Whole-cell pairwise—down-regulated in ΔcrtB**			
GO:0044264	Cellular polysaccharide metabolic process	GO_BiologicalProcess	12
GO:0009311	Oligosaccharide metabolic process	GO_BiologicalProcess	9
GO:0006629	Lipid metabolic process	GO_BiologicalProcess	23
GO:0008610	Lipid biosynthetic process	GO_BiologicalProcess	17
GO:0016798	Hydrolase activity, acting on glycosyl bonds	GO_MolecularFunction	9
GO:0004553	Hydrolase activity, hydrolyzing O-glycosyl compounds	GO_MolecularFunction	9
GO:0015926	Glucosidase activity	GO_MolecularFunction	5
GO:0090599	Alpha-glucosidase activity	GO_MolecularFunction	3
GO:0016903	Oxidoreductase activity, acting on the aldehyde or oxo group of donors	GO_MolecularFunction	9
GO:0019695	Choline metabolic process	GO_BiologicalProcess	4
GO:0031455	Glycine betaine metabolic process	GO_BiologicalProcess	4
GO:0006578	Amino-acid betaine biosynthetic process	GO_BiologicalProcess	4
GO:0019285	Glycine betaine biosynthetic process from choline	GO_BiologicalProcess	4
GO:0031456	Glycine betaine biosynthetic process	GO_BiologicalProcess	4
GO:0008802	Betaine-aldehyde dehydrogenase activity	GO_MolecularFunction	4
**Whole-cell pairwise—up-regulated in ΔcrtB**			
GO:0006260	DNA replication	GO_BiologicalProcess	4
GO:0050790	Regulation of catalytic activity	GO_BiologicalProcess	5
GO:1901698	Response to nitrogen compound	GO_BiologicalProcess	3
**MP pairwise—down-regulated in ΔcrtB**			
GO:0031241	Periplasmic side of cell outer membrane	GO_CellularComponent	4
GO:0031975	Envelope	GO_CellularComponent	21
GO:0030312	External encapsulating structure	GO_CellularComponent	16
GO:0030313	Cell envelope	GO_CellularComponent	19
GO:0044462	External encapsulating structure part	GO_CellularComponent	12
GO:0009279	Cell outer membrane	GO_CellularComponent	12
**DRM pairwise—down-regulated in ΔcrtB**			
GO:0048038	Quinone binding	GO_MolecularFunction	8
GO:0071944	Cell periphery	GO_CellularComponent	139
GO:0008104	Protein localization	GO_BiologicalProcess	17
GO:1904659	Glucose transmembrane transport	GO_BiologicalProcess	4
GO:0030001	Metal ion transport	GO_BiologicalProcess	15
GO:0022804	Active transmembrane transporter activity	GO_MolecularFunction	33
GO:0055085	Transmembrane transport	GO_BiologicalProcess	49
GO:0031224	Intrinsic component of membrane	GO_CellularComponent	104
GO:0005886	Plasma membrane	GO_CellularComponent	120
GO:0016021	Integral component of membrane	GO_CellularComponent	99
GO:0044459	Plasma membrane part	GO_CellularComponent	73
GO:0031226	Intrinsic component of plasma membrane	GO_CellularComponent	63
GO:0005887	Integral component of plasma membrane	GO_CellularComponent	61
GO:0031975	Envelope	GO_CellularComponent	31
GO:0098552	Side of membrane	GO_CellularComponent	14
GO:0030312	External encapsulating structure	GO_CellularComponent	23
GO:0030313	Cell envelope	GO_CellularComponent	26
GO:0044462	External encapsulating structure part	GO_CellularComponent	19
GO:0009279	Cell outer membrane	GO_CellularComponent	19
GO:0031230	Intrinsic component of cell outer membrane	GO_CellularComponent	7
GO:0031241	Periplasmic side of cell outer membrane	GO_CellularComponent	7
**DRM pairwise—up-regulated in ΔcrtB**			
GO:0005829	Cytosol	GO_CellularComponent	45
GO:0006090	Pyruvate metabolic process	GO_BiologicalProcess	6
GO:0043168	Anion binding	GO_MolecularFunction	28
GO:0032553	Ribonucleotide binding	GO_MolecularFunction	21
GO:0030554	Adenyl nucleotide binding	GO_MolecularFunction	18
GO:0032559	Adenyl ribonucleotide binding	GO_MolecularFunction	18
GO:0006082	Organic acid metabolic process	GO_BiologicalProcess	28
GO:0044283	Small molecule biosynthetic process	GO_BiologicalProcess	22
GO:0016053	Organic acid biosynthetic process	GO_BiologicalProcess	16
GO:0043436	Oxoacid metabolic process	GO_BiologicalProcess	28
GO:1901566	Organonitrogen compound biosynthetic process	GO_BiologicalProcess	25
GO:0019752	Carboxylic acid metabolic process	GO_BiologicalProcess	27
GO:0046394	Carboxylic acid biosynthetic process	GO_BiologicalProcess	16
**DSM pairwise—down-regulated in ΔcrtB**			
GO:1903509	Liposaccharide metabolic process	GO_BiologicalProcess	4
GO:0030312	External encapsulating structure	GO_CellularComponent	10
GO:0015850	Organic hydroxy compound transport	GO_BiologicalProcess	3
GO:0022838	Substrate-specific channel activity	GO_MolecularFunction	3
GO:0019725	Cellular homeostasis	GO_BiologicalProcess	3
GO:0048878	Chemical homeostasis	GO_BiologicalProcess	3
GO:0005783	Endoplasmic reticulum	GO_CellularComponent	3
GO:0046873	Metal ion transmembrane transporter activity	GO_MolecularFunction	4
GO:0030001	Metal ion transport	GO_BiologicalProcess	5
GO:0072511	Divalent inorganic cation transport	GO_BiologicalProcess	3
GO:0000041	Transition metal ion transport	GO_BiologicalProcess	4
GO:0070838	Divalent metal ion transport	GO_BiologicalProcess	3
**DSM pairwise—up-regulated in ΔcrtB**			
GO:0045229	External encapsulating structure organization	GO_BiologicalProcess	3
GO:0015293	Symporter activity	GO_MolecularFunction	3
GO:0022613	Ribonucleoprotein complex biogenesis	GO_BiologicalProcess	6
GO:0003723	RNA binding	GO_MolecularFunction	8
GO:0042254	Ribosome biogenesis	GO_BiologicalProcess	6
GO:0044446	Intracellular organelle part	GO_CellularComponent	11
GO:0070925	Organelle assembly	GO_BiologicalProcess	4
GO:0071826	Ribonucleoprotein complex subunit organization	GO_BiologicalProcess	4
GO:0006518	Peptide metabolic process	GO_BiologicalProcess	9
GO:0042273	Ribosomal large subunit biogenesis	GO_BiologicalProcess	3
GO:0043232	Intracellular non-membrane-bounded organelle	GO_CellularComponent	9
GO:0005840	Ribosome	GO_CellularComponent	9
GO:0019843	rRNA binding	GO_MolecularFunction	7
GO:0022618	Ribonucleoprotein complex assembly	GO_BiologicalProcess	4
GO:0034622	Cellular protein-containing complex assembly	GO_BiologicalProcess	4
GO:0043604	Amide biosynthetic process	GO_BiologicalProcess	9
GO:0043043	Peptide biosynthetic process	GO_BiologicalProcess	9
GO:0044391	Ribosomal subunit	GO_CellularComponent	9
GO:0006412	Translation	GO_BiologicalProcess	9
GO:0042255	Ribosome assembly	GO_BiologicalProcess	4
GO:0044445	Cytosolic part	GO_CellularComponent	9
GO:0000027	Ribosomal large subunit assembly	GO_BiologicalProcess	3
GO:0015934	Large ribosomal subunit	GO_CellularComponent	8
GO:0022626	Cytosolic ribosome	GO_CellularComponent	9
GO:0006364	rRNA processing	GO_BiologicalProcess	3
GO:0016072	rRNA metabolic process	GO_BiologicalProcess	3
GO:0022625	Cytosolic large ribosomal subunit	GO_CellularComponent	8

Whole gene ontology was performed using Blast2GO with a Blastp E-value hit filter of 1 × 10^–5^ and annotation cutoff value of 55 and a GO weight of 5. Using ClueGO, observed GO biological process were subjected to the right-sided hypergeometric enrichment test at medium network specificity selection and *p* value correction was performed using the Holm–Bonferroni step-down method.

In the whole cell pairwise comparisons, proteins belonging to lipid biosynthesis (GO:0008610), lipid metabolism (GO: 0006629) and oligosaccharide metabolism (GO:0009311) were less abundant in the Δ*crtB* mutant. Glycerophospholipids serve as the structural component of biological membranes and their alteration can affect physiology and adaptation^39^. Previously, we reported that the Δ*crtB* mutant shows a modest increase in phosphatidylethanolamine (PE) head groups and unsaturated fatty acids when compared to wild type cells^17^. This observation could be the consequence of down regulation of lysophospholipase (PMI39_02976), which is important for glycerophospholipid metabolism^40^, in the Δ*crtB* mutant. We also observed that a regulator of protease activity HflC, stomatin/ prohibitin superfamily-ybbK (2,511,379,369) appeared less abundant in the Δ*crtB* mutant, although the difference did not meet the criteria to be statistically significant (*p* value = 0.04 but fold change = 1.4). YbbK, encoded by PMI39_01287, belongs to the reggie (flotillin) superfamily, which includes eukaryotic flotillins and the bacterial homolog FlotP which was identified in *Bacillus anthracis* membrane microdomains^25,41,42^. The apparent reduction of YbbK in the carotenoid mutant is consistent with changes to microdomain organization, which may affect cellular functions such as protein signaling and transport. It is possible that the observed reduction of indole-3-acetic acid secretion and the decreased pellicle and biofilm formation observed in the Δ*crtB* mutant results from changes in membrane domain architecture.

Other proteins such as tyrosine kinase (2,511,379,927), cardiolipin synthase (2,511,380,815) and phytoene desaturase (2,511,381,490) were also less abundant in the Δ*crtB* mutant whole cell fraction. Notably, four undecaprenyl phosphate proteins (Locus tags- PMI39_03112, PMI39_03113, PMI39_03114, PMI39_03115) in an operon involved in amino sugar and nucleotide sugar metabolism were less abundant in the Δ*crtB* mutant^43^. Undecaprenyl phosphate is a 55-carbon polyisoprenoid lipid involved in bacterial cell wall biogenesis by functioning as a lipid carrier, trafficking sugar intermediates across the plasma membrane^44^. There is also growing evidence that polyisoprenoids increase membrane fluidity and ion permeability^45–48^. The downregulation of this operon may explain, at least in part, the observed decrease in membrane fluidity in the Δ*crtB* mutant^17^.

In the membrane fractions, several proteins with functional significance at the membrane were less abundant in the Δ*crtB* mutant (*p* ≤ 0.05 and FC ≥ 2). In particular, proteins belonging to envelope (GO:0030313), cell outer membrane (GO:0009279), membrane biogenesis, and cellular homeostasis were downregulated in the Δ*crtB* mutant. Homeostasis is important for living organisms to maintain internal stability and it includes iron and metal homeostasis, membrane lipid homeostasis, and pH homeostasis. For example, TonB (PMI39_04701), an outer membrane receptor for ferrienterochelin and colicins, which are important for iron homeostasis^49^, was less abundant in the Δ*crtB* DSM fraction.

### Transcriptional analyses of the Δ*crtB* mutant

**Figure 6:**
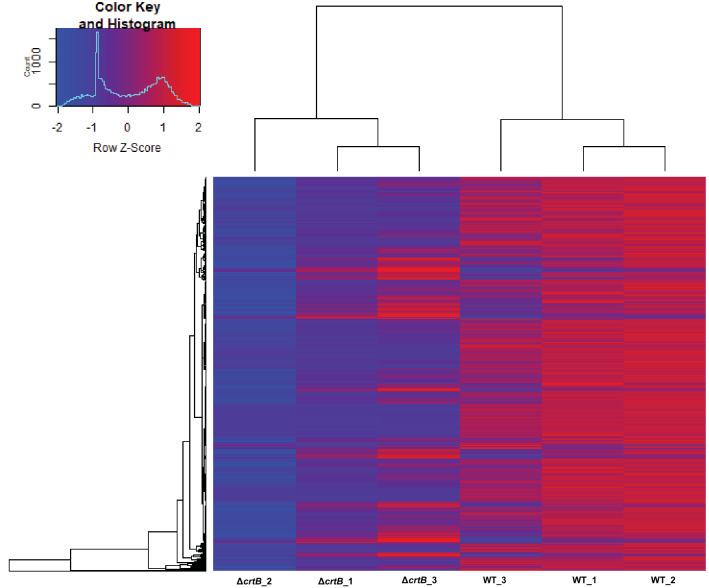
Clustered heatmap of gene expression in *Pantoea* sp. YR343 and the Δ*crtB* mutant. Hierarchical clustering was performed using absolute transcript counts. Genome wide transcriptional signatures indicated generally lower expression profiles in the Δ*crtB* mutant compared to the wildtype. Higher transcript levels are shown in red and lower transcript numbers are shown in blue. The heatmap was generated using gplots in Rstudio and scaled by row.

The differences observed in the membrane proteomes between wildtype and the Δ*crtB* mutant could be due to many factors, including differences in membrane protein insertion and stability, localization, or abundance. To better understand the basis for the observed differences, we performed transcriptional analyses to examine which gene products are transcriptionally regulated. Differentially expressed transcripts between the wildtype and Δ*crtB* mutant samples were identified using KBase tools as described in the methods^50^. Only 5 transcripts were significantly upregulated (*p* value ≤ 0.05 and FC ≥ 2), whereas 879 transcripts were significantly downregulated in the Δ*crtB* mutant (Supplemental Table 1). Heat maps representing the differential expression profile of wildtype and Δ*crtB* mutant are shown in Fig. 6. Detailed comparisons of the protein abundances and transcriptional regulation were performed for selected functional categories.

### Cell wall/membrane/envelope biogenesis

Proteins predicted to be involved in cell wall/membrane/envelope biogenesis (270 proteins total, COG category M) in *Pantoea* sp. YR343 were collected from the JGI IMG database. Table 2 lists all the proteins (*p* ≤ 0.005 and FC ≥ 1) involved in cell membrane biogenesis that were significantly differentially abundant in at least one fraction (56 total). Among these proteins, six undecaprenyl-phosphate (UDP) proteins belonging to peptidoglycan/lipopolysaccharide biosynthesis were found to be significantly less abundant in the Δ*crtB* mutant (PMI39_01550, PMI39_02251, PMI39_03115, PMI39_01848, PMI39_03114, PMI39_04793). UDP gene products are involved in exopolysaccharide secretion, cationic antimicrobial peptide resistance, lipid A biogenesis, and peptidoglycan synthesis^44^ and most were found in the DRM fractions. Transcript data for two of the UDP proteins, undecaprenyl-phosphate 4-deoxy-4-formamido-L-arabinose transferase (PMI39_03114) and UDP-4-amino-4-deoxy-L-arabinose-oxoglutarate aminotransferase (PMI39_03115), showed downregulation at the transcript level in the Δ*crtB* mutant (Table 2). Downregulation of these UDP genes may explain the observed differences in the peptidoglycan layer of the Δ*crtB* mutant in comparison to the wildtype^17^. Another protein, UDP-galactose-lipid carrier transferase (PMI39_01848), has a transmembrane domain and was found to be less abundant in the mutant DRM fraction. The gene encoding this protein is the first gene in a large operon that shows significant similarity to operons involved in EPS biosynthesis in the related plant-associated microbes *Erwinia amylovora* and *Pantoea stewartii*^51,52^. It is possible that the reduction in this protein decreases EPS production in the Δ*crtB* mutant, which may contribute to the defects associated with biofilm formation and plant colonization.

**Table 2:**
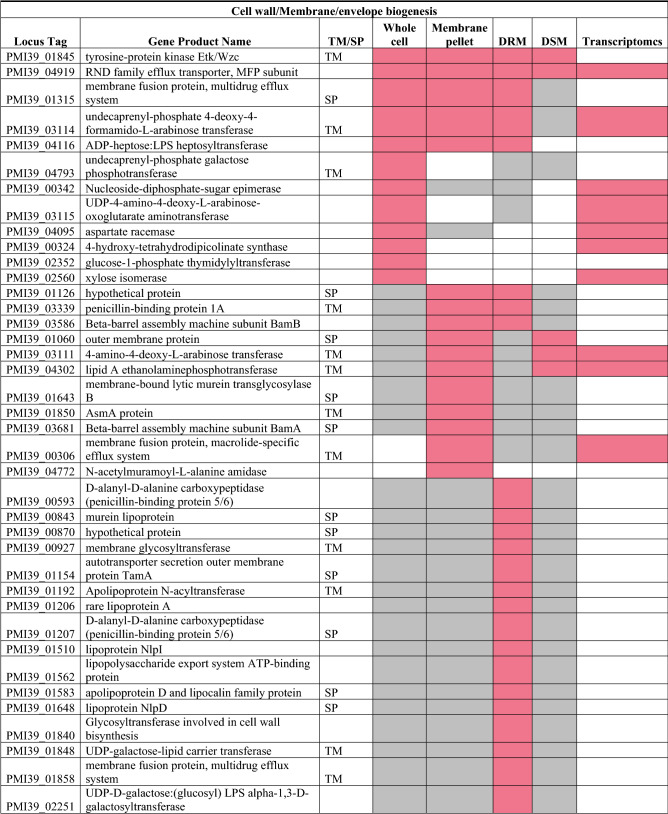

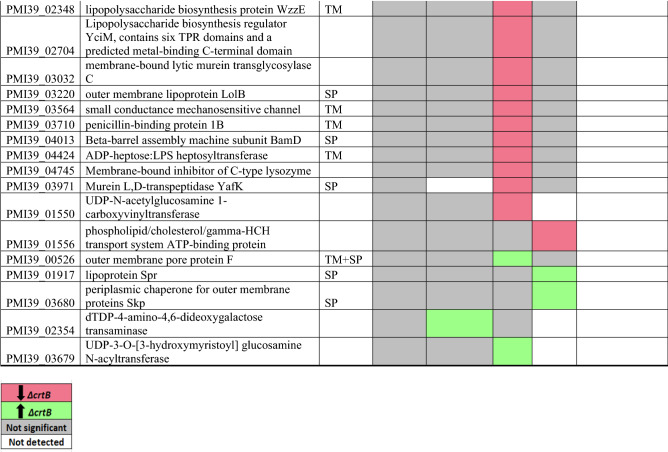
List of significantly differentially abundant proteins involved in cell wall/membrane/ envelope biogenesis.

Protein list from JGI for each COG category was matched with the proteomics dataset and only proteins that were significantly different in at least one fraction of the wildtype or *ΔcrtB* mutant are reported. TM/SP- proteins with transmembrane helices or signal peptide. Red: proteins that are significantly less abundant in the Δ*crtB* mutant, green: proteins that are significantly more abundant in the Δ*crtB* mutant, grey: non-significant proteins and white: proteins that are not detected.

Outer membrane proteins (OMP) are important for transport of metabolites and toxins, membrane biogenesis, and for bacterial resistance. The folding and insertion of several OMPs are carried out by BamA along with three lipoproteins: BamB, BamC, and BamE forming the BAM machine (beta-barrel assembly)^53,54^. Lipoproteins are peripherally anchored membrane proteins involved in cell division, chemotaxis, signal transduction and envelope stability^55–57^. Among the 4 Bam proteins, BamA (PMI39_03681) and BamB (PMI39_03586) were found to be less abundant in the Δ*crtB* mutant (Table 2). BamB was identified in both the membrane pellet and the DRM fraction. Studies have shown that BamB contains WD40 repeating units, thereby functioning as a scaffold protein in large multi-protein complexes^58^. It was also shown that the Bam complex increases the efficiency of folding of membrane proteins such as OmpA and EspP^59^. We also found that the Skp protein (PMI39_3680) was more abundant in the Δ*crtB* mutant DSM fraction. Skp is a multivalent periplasmic chaperone preventing misfolding and aggregation of OMPs, such as OmpA, during transit from the inner to the outer membranes^60^. The changes in membrane fluidity, lipid content, and the lack of carotenoids in the Δ*crtB* mutant may influence assembly of the Bam complex at the outer membrane, leading to misfolding of other OMPs, but not affect localization or function of the periplasmic Skp protein. Thus, the increased abundance of Skp in the Δ*crtB* mutant may be a compensatory mechanism to maintain proper folding of proteins to protect the integrity of the cell.

### Cell motility (N)

**Figure 7:**
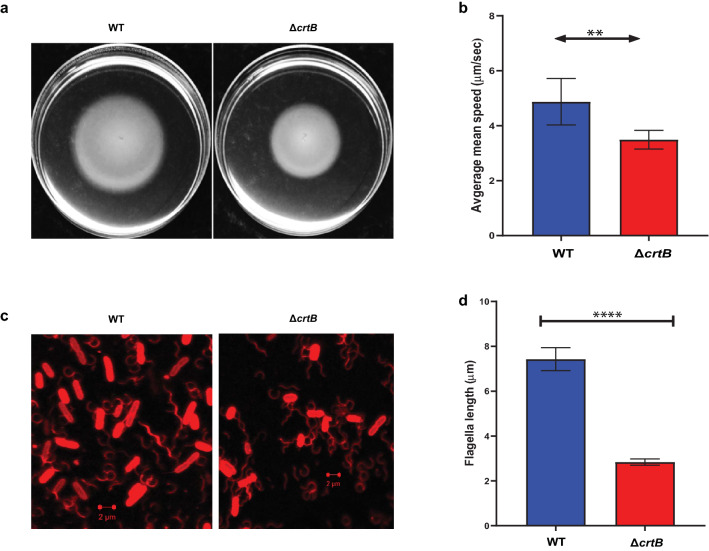
Loss of carotenoids affects bacterial cell motility. (**a**) Swimming motility of wildtype and the Δ*crtB* mutant on LB plates with 0.3% agar. Cells were inoculated at the center of the plate from an overnight culture and photographed after 16 h incubation at 28 °C. (**b**) Cells from motility plates were grown to OD_600_ of 0.5 and swimming motility videos of 10 biological replicates were collected and processed by ImageJ. (**c**) Flagella staining with Alexa Fluor 594 carboxylic acid succinimidyl ester was carried out on log-phase cells and imaged using confocal microscopy. (**d**) Flagellar length measurement (μm) of 30 wildtype and Δ*crtB* mutant cells using ImageJ. Statistical significance was detected by t-test *p* ≤ 0.0001 (****); *p* ≤ 0.001 (***); *p* ≤ 0.01 (**) and *p* ≤ 0.05 (*).

Beyond the defects in IAA secretion, biofilm formation, and root colonization previously reported^29^, we also observed that the Δ*crtB* mutant appeared to be less motile than wildtype on swimming motility plates (Fig. 7a). To further characterize this defect, we compared motility patterns of wildtype and mutant cells by microscopy. We found that the average mean speed was 3.5 μm/s for the Δ*crtB* mutant which was significantly reduced compared to 4.9 μm/s for wild type cells (Fig. 7b). Moreover, flagella staining and quantification using ImageJ indicated that the Δ*crtB* mutant had significantly shorter flagella when compared to wildtype (Fig. 7c). The average flagellar length for the wildtype cells were 7.4 μm whereas the Δ*crtB* mutant flagella measured 2.8 μm (Fig. 7d). To help explain the motility defect, we examined the 92 proteins predicted to be involved in cell motility, of which 18 were differentially abundant in the mutant compared to wildtype (Table 3). Among the 28 proteins that form the flagellar complex^61^, only 3 proteins: flagellar FliL (PMI39_02182) and two flagellar hook-associated protein 2 (PMI39_02605 and PMI39_02159) were significantly less abundant in the Δ*crtB* mutant and only one of these, PMI39_02605, was significantly downregulated based on the transcriptomic data (Table 3). In *Salmonella* and *E. coli*, it was shown that FliL interacts closely with stators and the MS ring, ensuring delivery of higher torque, leading to increased motility^62^. In the absence of the FliL protein, single motors have been shown to rotate at lower speeds^62,63^. It is possible that the changes in membrane lipid composition and/or organization affect assembly or function of the flagellar motor apparatus, leading to the observed motility defects.

**Table 3:**
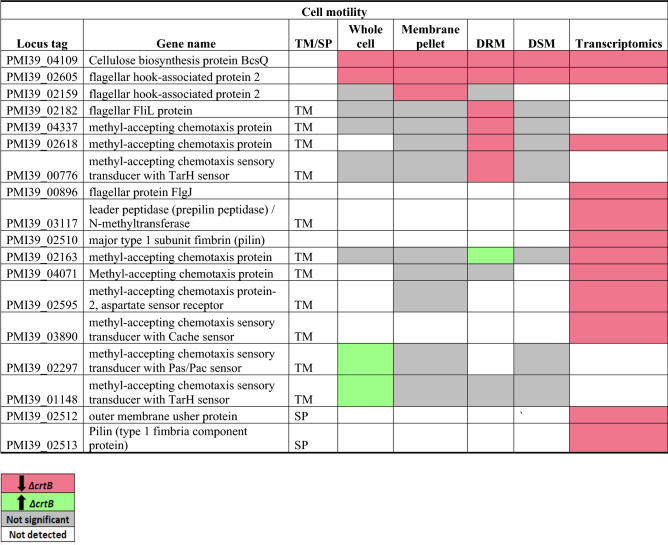
List of significantly differentially abundant proteins involved in cell motility.

Protein list from JGI for each COG category was matched with the proteomics dataset and only proteins that were significantly different in at least in one fraction in the wildtype or Δ*crtB* mutant are reported. TM/SP- proteins with transmembrane helices or signal peptide. Red: proteins that are significantly less abundant in the Δ*crtB* mutant, green: proteins that are significantly more abundant in the Δ*crtB* mutant, grey: non-significant proteins and white: proteins that are not detected.

In addition to the proteins involved in the flagellar motor apparatus, we also found differences in protein abundance and transcriptional regulation of several methyl-accepting chemotaxis proteins (MCP) in the Δ*crtB* mutant compared to wild type (Table 3). MCPs undergo reversible methylation in response to changes in the concentration of attractants or repellents in their environment^64^. Interestingly, two MCP proteins, encoded by PMI39_02297 and PMI39_01148, were found to be more abundant in the Δ*crtB* whole cell fraction, whereas the MCP encoded by PMI39_02163 was found to be more abundant in the Δ*crtB* DRM fraction. This increased abundance in the Δ*crtB* mutant did not appear to be due to transcriptional upregulation (Table 3). Additional experiments are needed to distinguish whether these proteins are differentially localized, more stable, or perhaps more easily extracted from the Δ*crtB* mutant.

### Lipid transport and metabolism (I)

In *Pantoea* sp. YR343, 150 proteins are found in the lipid transport and metabolism COG category I, of which 26 proteins were found to be significantly abundant in at least one fraction (Table 4). Two choline dehydrogenases (encoded by PMI39_02890 and PMI39_00318) were significantly more abundant in all or most fractions of the Δ*crtB* mutant. Surprisingly, however, these genes were transcriptionally downregulated (Table 4). Choline dehydrogenase catalyzes the first step in glycine betaine synthesis to produce the final compound betaine, an effective osmoprotectant^65,66^. It is possible that the lipid composition or membrane organization in the carotenoid mutant promotes choline dehydrogenase protein stability or, alternatively, promotes its efficient extraction. Other proteins such as lysophospholipase (PMI39_01261, PMI39_04916) and NAD(P) dependent dehydrogenasese (PMI39_04227, PMI39_04693, PMI39_04133) were also more abundant in the Δ*crtB* mutant.

**Table 4:**
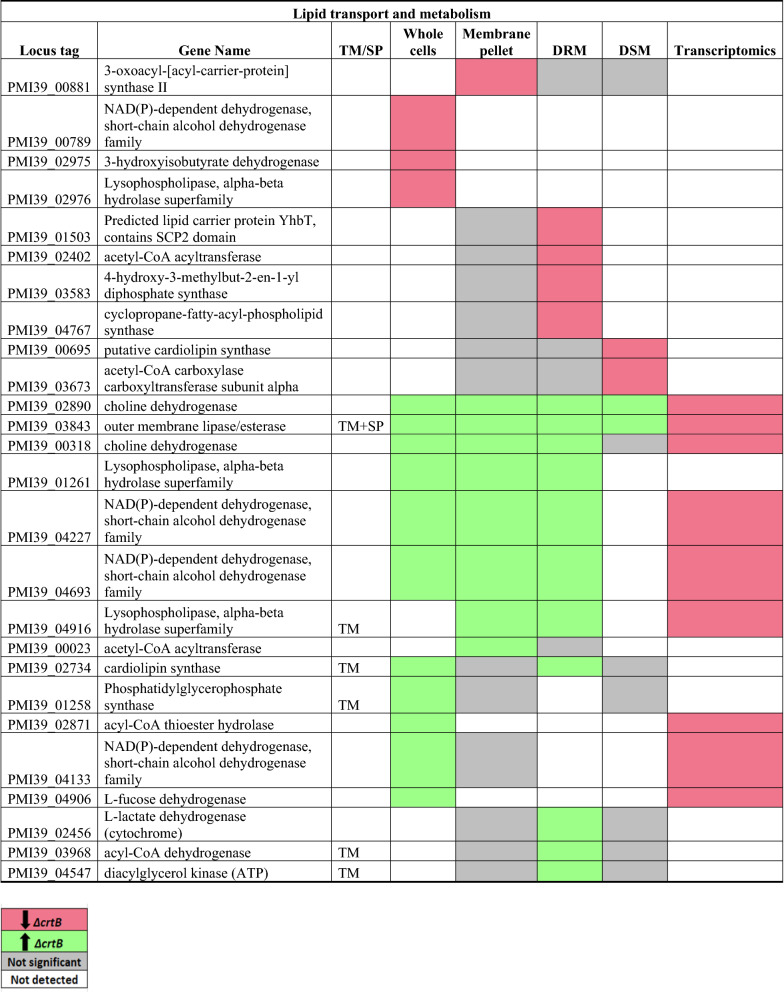
List of significantly differentially abundant proteins involved in lipid transport and metabolism.

Protein list from JGI for each COG category was matched with the proteomics dataset and only proteins that were significantly different in at least in one fraction in the wildtype or Δ*crtB* mutant are reported. TM/SP- proteins with transmembrane helices or signal peptide. Red: proteins that are significantly less abundant in the Δ*crtB* mutant, green: proteins that are significantly more abundant in the Δ*crtB* mutant, grey: non-significant proteins and white: proteins that are not detected.

Cyclopropane-fatty-acyl-phospholipid synthase (PMI39_04767) and predicted lipid carrier protein YhbT, containing a SCP2 domain (PMI39_01503), were less abundant in the Δ*crtB* DRM fractions. The physiological role of YhbT has not yet been identified, but it contains the sterol carrier protein 2 domain (SCP2), suggesting a role in lipid and sterol transport^67^.

### Signal transduction mechanism (T)

Bacterial signal transduction networks regulate sensing and responses to environmental and intracellular parameters. In *Pantoea* sp. YR343, 235 proteins are predicted to be involved in signal transduction based on the COG category T (Table 5). Among these proteins, only 32 proteins were found to be differentially abundant in at least one fraction. In our data, we observed an abundance of OmpR family proteins^68^, including the phosphate regulon response regulator OmpR (PMI39_03347) in the Δ*crtB* DRM fractions. OmpR, along with its histidine kinase partner EnvZ, are important for osmotic tolerance, virulence and motility in *Acinetobacter baumanii*^69–71^. In *E. coli*, OmpR and EnvZ regulate OmpF and OmpC proteins that are essential for responding to environmental signals.

**Table 5:**
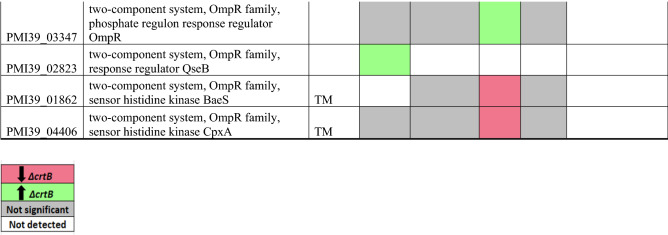

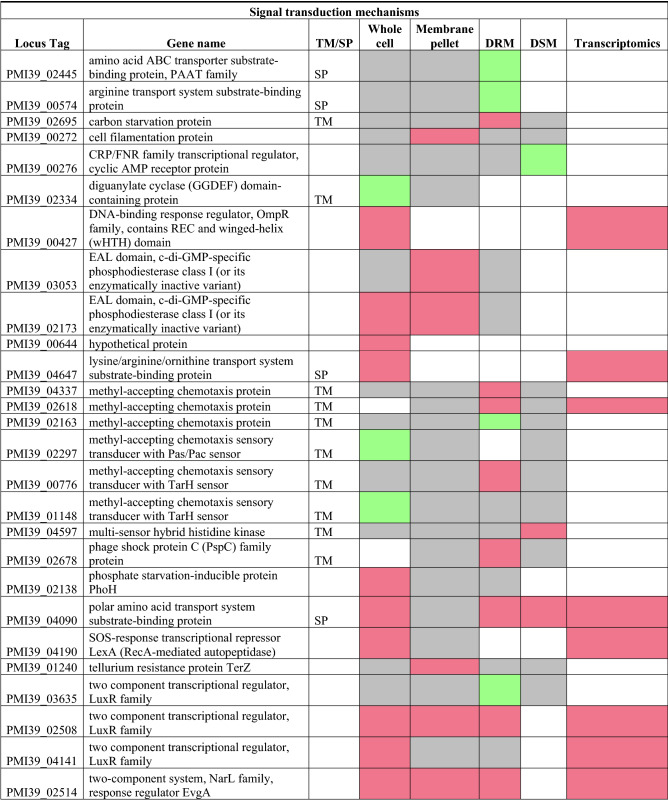
List of significantly differentially abundant proteins involved in signal transduction mechanisms.

Protein list from JGI for each COG category was matched with the proteomics dataset and only proteins that were significantly different in at least in one fraction in the wildtype or Δ*crtB* mutant are reported. TM/SP- proteins with transmembrane helices or signal peptide. Red: proteins that are significantly less abundant in the Δ*crtB* mutant, green: proteins that are significantly more abundant in the Δ*crtB* mutant, grey: non-significant proteins and white: proteins that are not detected.

## Conclusion

The deletion of carotenoids in the Δ*crtB* mutant leads not only to increased sensitivity to oxidative stress, but also to defects in IAA secretion, pellicle and biofilm formation, motility, and root colonization. In addition to the differences in lipid composition and membrane fluidity previously reported^17^, the loss of carotenoids also results in changes to the proteome of the Δ*crtB* mutant compared to wildtype. We report a detailed proteome analysis comparing the wildtype and Δ*crtB* mutant focusing on changes in membrane protein distribution and abundance. Consistent with the observed phenotypes in the mutant, we found that several classes of proteins belonging to membrane biogenesis, signal transduction, and cell motility were affected in the Δ*crtB* mutant. The most dramatic changes to the proteome were observed in the DRM fraction, which is consistent with the idea that the DRM fraction represents membrane microdomains and the presence of cholesterol (in eukaryotes) or carotenoids and hopanoids (in prokaryotes) is vital to the organization of these domains^72^. In the absence of carotenoids, these microdomains may be unstable or have a change in membrane thickness which, in turn, affects protein insertion, stability, or recruitment. These data underscore the importance of bacterial membrane organization for cellular functions such as secretion, motility, and signaling.

## Methods

### Bacterial strains and growth conditions

*Pantoea sp.* YR343 and Δ*crtB* cells were grown in Luria–Bertani broth (per 1 L, 10 g Bacto-tryptone, 10 g NaCl, 5 g yeast extract) medium at 28 °C with shaking to OD_600_ of 1 (stationary phase). The Δ*crtB* mutant was constructed as described^29^.

### Isolation of whole cell, crude membrane fraction, detergent resistant membrane and detergent sensitive membrane fractions of *Pantoea* sp. YR343 and Δ*crtB* cells

To isolate different cell fractions, we used a modified version of the method described by Lopez^18^. Briefly, cells were grown in 500 mL of LB media overnight at 28 °C with vigorous shaking. Cells were collected by centrifugation (3000*g* for 12mins) and washed thrice in phosphate buffered saline (PBS). Cells were collected at this stage for whole cell proteomic analysis and stored at − 20 °C. Next, Buffer H (20 mM 4-(2-hydroxyethyl)-1-piperazineethanesulfonic acid (HEPES [pH 8], 20 mM NaCl, 1 mM dithiothreitol [DTT], 1 mM phenylmethylsulfonyl fluoride [PMSF]), lysozyme (1 mg/mL), PMSF (100 μM), and DNase I was added to the washed cells. Cells were disrupted using French press followed by a short centrifugation to eliminate cell debris. The membrane fraction was precipitated by ultracentrifugation (100,000×*g* for 1 h at 4 °C). The resulting cell pellet was resuspended in Buffer H with 10% glycerol. At this stage, a fraction of the membrane pellet was collected.

To isolate DRM and DSM fractions, the membrane pellet was incubated for 30 min at 4 °C with lysis and separation buffer (CelLytic MEM protein extraction kit from Sigma Aldrich). After incubation, the membrane pellet was mixed 1:1 with 80% sucrose and carefully overlaid with 20% sucrose. Using a swinging bucket rotor, separation was carried out at 100,000×*g* at 4 °C for 16 h. The DRM and DSM fractions were collected and stored at − 20 °C for proteomic analysis.

### Protein extraction and digestion

Cell pellets were suspended in sodium dodecyl sulfate (SDS) lysis buffer (2% in 100 mM of NH_4_HCO_3_, 10 mM DTT). Samples were physically disrupted by bead beating (0.15 mm) at 8,000 rpm for 5 min. Crude lysates were boiled for 5 min at 90 °C. Cysteines were blocked to avoid disulfide bridge reformation by adjusting each sample to 30 mM IAA and incubating in the dark for 15 min at room temperature. Proteins were precipitated using a chloroform/methanol/water extraction. Dried protein pellets were resuspended in 2% sodium deoxycholate (SDC) (100 mM NH_4_HCO_3_) and protein amounts were estimated by performing a BCA assay (Pierce Biotechnology). In general, membrane fractions from the mutant showed a reduced protein concentration compared to wild type (MP: 3.7 mg/mL (wt) and 2.8 mg/mL (Δ*crtB*); DSM: 858 µg/mL (wt) and 845 µg/mL (Δ*crtB*); DRM: 165 µg/mL (wt) and 138 µg/mL (Δ*crtB*)). For each sample, an aliquot of approximately 500 µg of protein was digested via two aliquots of sequencing-grade trypsin (Promega, 1:75 [w:w]) at two different sample dilutions, (overnight) followed by incubating 3 h at 37 °C. The peptide mixture was adjusted to 0.5% formaldehyde (FA) to precipitate SDC. Hydrated ethyl acetate was added to each sample at a 1:1 [v:v] ratio three times to effectively remove SDC. Samples were then placed in a SpeedVac Concentrator (Thermo Fischer Scientific) to remove ethyl acetate and further concentrate the sample. The peptide-enriched flow through was quantified using the BCA assay, desalted on RP-C18 stage tips (Pierce Biotechnology) and then stored at − 80 °C prior to LC–MS/MS analysis.

### LC–MS/MS

All samples were analyzed on a Q Exactive Plus mass spectrometer (Thermo Fisher Scientific) coupled with a Proxeon EASY-nLC 1200 liquid chromatography (LC) pump (Thermo Fisher Scientific) as previously described^73^. In brief, peptide mixtures were separated on a 75 μm inner diameter microcapillary column packed with 30 cm of Kinetex C18 resin (1.7 μm, 100 Å, Phenomenex). For each peptide mixture, a 2 μg aliquot was loaded in buffer A (0.1% formic acid, 2% acetonitrile) and eluted with a linear 150 min gradient of 2–20% of buffer B (0.1% formic acid, 80% acetonitrile), followed by an increase in buffer B to 30% for 10 min, another increase to 50% buffer for 10 min and concluding with a 10 min wash at 98% buffer A. The flow rate was kept at 200 nL/min. Mass spectra data was acquired with the Thermo Xcalibur software version 2.2, and a topN method where N could be up to 15 was employed for data-dependent acquisition^73^.

### Peptide identification and protein inference

MS raw data files were searched against the *Pantoea sp.* YR343 FASTA database to which common contaminate proteins had been added. A decoy database, consisting of the reversed sequences of the target database, was appended to discern the false-discovery rate (FDR) at the spectral level. For standard database searching, the peptide fragmentation spectra (MS/MS) were analyzed by the Crux pipeline v3.0^74^. The MS/MS were searched using the Tide algorithm^75^ and was configured to derive fully-tryptic peptides using default settings except for the following parameters: allowed clip nterm-methionine, a precursor mass tolerance of 10 parts per million (ppm), a static modification on cysteines (iodoacetamide; + 57.0214 Da), and dynamic modifications on methionine (oxidation; 15.9949). The results were processed by Percolator^76^ to estimate *q* values. Peptide spectrum matches (PSMs) and peptides were considered identified at a *q* value < 0.01. Across the entire experimental dataset, proteins were required to have at least 2 distinct peptide sequences and 2 minimum spectra per protein.

### Protein quantification

For label-free quantification, MS1-level precursor intensities were derived from MOFF^77^ using the following parameters: 10 ppm mass tolerance, retention time window for extracted ion chromatogram was 3 min, time window to get the apex for MS/MS precursor was 30 s. Protein intensity-based values, which were calculated by summing together quantified peptides, normalized by dividing by protein length and then LOESS and median central tendency procedures were performed on log2-transformed data using the freely available software Perseus (. Missing values were replaced by random numbers drawn from a normal distribution (width = 0.3 and downshift = 2.8).

### Statistical analysis for differential abundances

For this study, we performed ANOVA with post-hoc Tukey’s test to identify differential protein abundances across the wildtype *Pantoea* sp. YR343 dataset comparisons or Δ*crtB* mutant dataset comparisons and protein abundances were considered to have a significant change in abundance for *p* values < 0.05 and at least one absolute value of log2 fold-change differences > 1. To identify differential protein abundances between wildtype and Δ*crtB* fractions, we performed a Student’s t-test for the pairwise comparisons. A protein was categorized as having a significant abundance difference if it passed a significance threshold requiring a *p* value < 0.05 and absolute value of log2 fold-change difference > 1. Hierarchical clustering (one-way; Fast Ward method) was performed to identify differential abundance patterns.

### Gene ontology enrichment

Gene ontology (GO) term annotation was performed using Blast2GO^38^ with a blastp E-value hit filter of 1 × 10^–5^, an annotation cutoff value of 55 and a GO weight of 5. Using the Cytoscape^79^ plugin ClueGO^80^, observed GO biological processes were subjected to the right-sided hypergeometric enrichment test at medium network specificity selection and p-value correction was performed using the Holm–Bonferroni step-down method^81^. For each cluster, we required a minimum of 3 and a maximum of 8 selected GO tree levels, and each cluster was set to include a minimum of 3- 4% of genes associated with each term. The GO terms at adjusted p < 0.05 were considered significantly enriched.

### RNA extraction, sequencing and analysis

Wild type and Δ*crtB* cells were grown to stationary phase (OD_600_ = 1). RNA was extracted using RNeasy mini kit (QIAGEN, Valencia, CA) following manufacturer’s instructions and quantified using Nanodrop (Thermo Scientific). Sequencing was carried out by GENEWIZ Next Generation Sequencing Services. Transcript analysis was carried out using KBase^50^ (https://kbase.us/). KBase and its tools were used to generate the sample set, align and assemble reads to the genome, and identify differentially abundant genes between wild type and Δ*crtB*.

### Motility assays

To compare the swimming motility function of *Pantoea* sp. YR343 and Δ*crtB* cells, cells were grown overnight with shaking (250 rpm) in LB medium at 28 °C. Swimming motility was examined on LB containing 0.3% w/v agar. A 5 μL aliquot of cells were inoculated in the center of the plate and incubated at 28 °C for 18 h. Live cell imaging of bacterial motility was measured using a Nikon Eclipse T*i*-U inverted microscope. Cells from motility plates were inoculated in R2A media overnight at 28 °C with shaking (250 rpm). Next day, cells were reinoculated in R2A media and grown to an OD_600_ of 0.5. A 20 µL aliquot of cells were placed on a coverslip and 10 s videos were captured using NIS-Elements imaging software. Trajectories and velocities (pixels/frame) of *Pantoea* sp. YR343 and Δ*crtB* cells were calculated with the “TrackMate” plugin (https://imagej.net/TrackMate).

### Flagella staining

Flagella staining was carried out using a protocol adapted from Turner et al.^82^. Briefly, *Pantoea* sp. YR343 and Δ*crtB* cells from swimming plates were inoculated overnight in R2A medium at 28 °C with shaking (250 rpm). Next day, cells were diluted 1:10 in fresh R2A medium and grown to OD_600_ of 0.5. Motility of the culture was confirmed using a confocal microscope. Cells were collected by centrifugation (2000*g, 3 min) and washed three times in buffer (0.01 M KPO_4_, 0.067 M NaCl, 10^−4^ M (Ethylenediaminetetraacetic acid (EDTA) [pH 7.0]). Alexa Fluor 594 carboxylic acid succinimidyl ester (ThermoFisher Scientific) was added to the concentrated bacterial suspension and incubated in the dark for 1 h. Cells were then washed three times with buffer containing Brij 35 (10^−4^%) and 0.4% glucose. Concentrated cells were then placed on an agarose pad (1% agarose in phosphate buffered saline) and imaged using a Zeiss LSM 710 confocal microscope. Flagellar length of 30 wildtype and Δ*crtB* cells were measured using ImageJ. The data are represented as the mean flagellar length in µm ± SE calculated using unpaired t-test.

## Supplementary Information

Supplementary Information.

## Data Availability

All proteomics mass spectrometry data collected in this study was deposited at the ProteomeXchange Consortium via the MASSIVE repository under the project identifier MSV000085068.
