# Supplementary material for: A carotenoid-deficient mutant of the plant-associated microbe Pantoea sp. YR343 displays an altered membrane proteome
Source: Sci Rep. 2020 Sep 11;10:14985. doi: 10.1038/s41598-020-71672-w (PMC7486946; doi:10.1038/s41598-020-71672-w)
Supplement: Supplementary file 1 — Supplementary Information. [file 41598_2020_71672_MOESM1_ESM.pdf]

Supplemental Data

A carotenoid-deficient mutant of the plant-associated microbe *Pantoea* sp. YR343 displays an altered membrane proteome

Sushmitha Vijaya Kumar, Paul E. Abraham, Gregory B. Hurst, Karuna Chourey, Amber N. Bible, Robert L. Hettich, Mitchel J. Doktycz, Jennifer L. Morrell-Falvey

Supplementary Table 1. A list of genes that are significantly upregulated or downregulated in the Δ*crtB* mutant.

Supplementary Table 1.

A. List of transcripts upregulated in *ΔcrtB* mutant cells. Transcripts with log2_FC cutoff of ≥2 and p-value ≤0.05 are shown below.

Transcripts upregulated in *ΔcrtB* mutant cells			
**Locus tag**	**Gene Product Name**	**log2_FC**	**p_value**
PMI39_00250	ribokinase	2.66904555	9.79E-74
PMI39_01552	anti-sigma B factor antagonist	3.20467	0.005816
PMI39_04409	glycerol-3-phosphate dehydrogenase	10.4384142	3.42E-285
PMI39_04621	metal resistance protein	2.11354001	1.75E-22
PMI39_04900	elongation factor G	2.50191556	3.99E-146

B. List of transcripts downregulated in *ΔcrtB* mutant cells. Transcripts with log2_FC cutoff of ≥2 and p-value ≤0.05 are shown below.

Transcripts upregulated in *ΔcrtB* mutant cells			
**Locus tag**	**Gene Product Name**	**log2_FC**	**p_value**
PMI39_00128	holo-[acyl-carrier-protein] synthase	-2.798236	0.009270478
PMI39_00205	Site-specific DNA recombinase	-4.382789	5.17E-116
PMI39_00206	putatice virulence related protein PagC	-4.44461	1.47E-106
PMI39_00207	putatice virulence related protein PagC	-4.216057	1.23E-102
PMI39_00209	hypothetical protein	-4.33775	8.25E-120
PMI39_00210	hypothetical protein	-4.465043	1.45E-92
PMI39_00211	Uncharacterized homolog of phage Mu protein gp47	-4.257536	4.77E-145
PMI39_00212	hypothetical protein	-4.601179	4.59E-96
PMI39_00213	hypothetical protein	-4.344751	2.15E-111
PMI39_00214	hypothetical protein	-4.288323	5.76E-146
PMI39_00215	hypothetical protein	-4.280771	2.46E-83
PMI39_00216	Protease subunit of ATP-dependent Clp proteases	-4.080806	2.17E-176
PMI39_00217	Protein of unknown function (DUF2767)	-4.818347	9.23E-218
PMI39_00218	hypothetical protein	-4.377319	6.68E-123
PMI39_00219	SIR2-like domain-containing protein	-3.85704	6.41E-201
PMI39_00220	hypothetical protein	-3.920508	2.73E-103
PMI39_00221	Protein-disulfide isomerase	-4.425231	2.69E-111
PMI39_00222	hypothetical protein	-4.720376	1.91E-158
PMI39_00223	hypothetical protein	-4.4777	1.88E-137
PMI39_00224	hypothetical protein	-4.638454	4.33E-130
PMI39_00225	hypothetical protein	-4.598449	2.96E-104
PMI39_00226	hypothetical protein	-4.673895	2.51E-128

PMI39_00227	hypothetical protein	-4.555286	1.48E-105
PMI39_00228	hypothetical protein	-5.099756	2.05E-79
PMI39_00229	hypothetical protein	-4.853886	2.45E-28
PMI39_00230	hypothetical protein	-4.54698	1.82E-58
PMI39_00231	protein of unknown function (DUF4055)	-4.403778	1.64E-85
PMI39_00232	hypothetical protein	-4.207735	3.04E-184
PMI39_00233	phage terminase small subunit	-3.99862	6.20E-145
PMI39_00234	hypothetical protein	-4.101221	2.43E-165
PMI39_00235	Colicin immunity protein / pyocin immunity protein	-3.385193	5.38E-99
PMI39_00237	lysozyme	-4.478866	6.13E-84
PMI39_00238	Bacteriophage P21 holin S	-3.741967	1.64E-58
PMI39_00239	Antitermination protein	-4.216044	3.31E-108
PMI39_00240	Response regulator	-3.901708	3.75E-82
PMI39_00241	hypothetical protein	-3.85888	9.65E-89
PMI39_00242	MFS transporter, DHA2 family, multidrug resistance protein	-3.952174	1.45E-163
PMI39_00243	DNA polymerase V	-4.1174	1.74E-145
PMI39_00244	DNA polymerase V	-3.873906	1.33E-102
PMI39_00245	hypothetical protein	-3.713643	3.79E-99
PMI39_00246	hypothetical protein	-3.963675	1.64E-150
PMI39_00247	hypothetical protein	-3.835641	3.52E-148
PMI39_00293	hypothetical protein	-3.748404	1.55E-125
PMI39_00294	hypothetical protein	-4.038532	7.07E-87
PMI39_00295	hypothetical protein	-4.022854	3.69E-83
PMI39_00296	transcriptional regulator, TetR family	-4.062069	3.40E-110
PMI39_00297	N-ethylmaleimide reductase	-3.782178	2.90E-77
PMI39_00298	hypothetical protein	-3.742426	5.44E-85
PMI39_00299	Short-chain dehydrogenase	-3.772589	4.85E-143
PMI39_00302	ATPase components of ABC transporters with duplicated ATPase domains	-3.620675	1.81E-147
PMI39_00303	Transmembrane transcriptional regulator (anti-sigma factor RsiW)	-3.470316	2.22E-87
PMI39_00304	RNA polymerase sigma-70 factor, ECF subfamily	-3.602549	1.78E-114
PMI39_00305	TPR repeat-containing protein	-3.757199	7.23E-130
PMI39_00306	membrane fusion protein, macrolide-specific efflux system	-3.504737	2.77E-109
PMI39_00307	macrolide transport system ATP-binding/permease protein	-3.567919	1.40E-128
PMI39_00308	transcriptional regulator, RpiR family	-3.584949	4.69E-77
PMI39_00309	PTS system, maltose and glucose-specific IIC component	-3.501648	1.54E-113
PMI39_00310	N-acylglucosamine-6-phosphate 2-epimerase	-3.669272	1.95E-131
PMI39_00311	choline/glycine/proline betaine transport protein	-3.737722	5.92E-192
PMI39_00312	transcriptional regulator, TetR family	-3.334475	4.64E-105

PMI39_00317	NAD-dependent aldehyde dehydrogenases	-3.098981	1.07E-86
PMI39_00318	choline dehydrogenase	-3.521412	5.62E-187
PMI39_00319	Cyanate permease	-4.192715	8.20E-72
PMI39_00320	transcriptional regulator, IclR family	-3.861767	6.06E-119
PMI39_00321	Mannose-6-phosphate isomerase, cupin superfamily	-3.961022	1.29E-79
PMI39_00322	Protein of unknown function (DUF1471)	-5.005554	1.18E-268
PMI39_00323	Protein of unknown function (DUF1471)	-5.514058	0
PMI39_00324	4-hydroxy-tetrahydrodipicolinate synthase	-3.818062	1.27E-82
PMI39_00325	hypothetical protein	-4.178008	1.49E-214
PMI39_00326	outer-membrane receptor for ferric coprogen and ferric-rhodotorulic acid	-4.376577	1.04E-222
PMI39_00327	probable lipoprotein NlpC	-4.23914	1.49E-242
PMI39_00328	Isopenicillin N synthase	-4.71465	6.76E-75
PMI39_00329	NitT/TauT family transport system substrate-binding protein	-4.677395	1.65E-64
PMI39_00330	Cytosine/adenosine deaminase	-3.521239	3.35E-57
PMI39_00331	drug resistance transporter, EmrB/QacA subfamily	-4.086986	8.06E-181
PMI39_00332	transcriptional regulator, TetR family	-3.564052	2.84E-89
PMI39_00333	Peroxiredoxin	-4.220121	6.78E-92
PMI39_00334	thiol:disulfide interchange protein DsbD	-3.83058	2.33E-161
PMI39_00335	RNA polymerase sigma-70 factor, ECF subfamily	-3.96119	1.15E-57
PMI39_00336	Protein of unknown function (DUF1109)	-4.037772	3.70E-121
PMI39_00337	3-dehydro-L-gulonate 2-dehydrogenase	-4.03534	3.69E-215
PMI39_00338	oligogalacturonide transporter	-4.289066	6.25E-233
PMI39_00339	Protein of unknown function (DUF1471)	-3.399263	3.82E-49
PMI39_00340	Multidrug resistance efflux pump	-4.374927	5.58E-159
PMI39_00341	Protein of unknown function (DUF3302)	-4.52532	1.87E-180
PMI39_00342	Nucleoside-diphosphate-sugar epimerase	-3.755068	1.02E-81
PMI39_00343	DNA-binding transcriptional regulator, LysR family	-3.964928	1.23E-172
PMI39_00372	Uncharacterized membrane protein YhdT	-8.159252	3.22E-06
PMI39_00374	Chloride channel protein EriC	-4.034067	3.04E-124
PMI39_00375	Ferritin-like metal-binding protein YciE	-4.176782	2.25E-114
PMI39_00376	hypothetical protein	-4.163163	4.61E-76
PMI39_00377	hypothetical protein	-4.621629	3.07E-78
PMI39_00379	Cellobiose phosphorylase	-4.033398	5.70E-161
PMI39_00380	Cytosine/adenosine deaminase	-3.97627	8.31E-113
PMI39_00384	AraC-type DNA-binding protein	-3.667001	2.74E-69
PMI39_00385	DNA-binding transcriptional regulator, LysR family	-4.106883	1.58E-124
PMI39_00386	Glyoxylase, beta-lactamase superfamily II	-4.071537	1.30E-266
PMI39_00387	Permease of the drug/metabolite transporter (DMT) superfamily	-4.539063	3.81E-198

PMI39_00388	2-polyprenyl-6-methoxyphenol hydroxylase	-4.284708	1.27E-149
PMI39_00389	AraC-type DNA-binding protein	-4.074309	4.94E-80
PMI39_00390	Short-chain dehydrogenase	-4.259309	2.44E-39
PMI39_00391	hypothetical protein	-4.391799	2.35E-116
PMI39_00392	hypothetical protein	-4.095063	9.70E-164
PMI39_00393	Nicotinamidase-related amidase	-4.358591	1.06E-171
PMI39_00394	hypothetical protein	-4.398133	5.28E-97
PMI39_00395	DNA-binding transcriptional regulator, LacI/PurR family	-4.118139	2.14E-177
PMI39_00396	resistance to homoserine/threonine (RhtB) family protein	-4.413681	1.87E-123
PMI39_00397	Uncharacterized conserved protein YbaA, DUF1428 family	-4.347959	3.09E-130
PMI39_00398	hypothetical protein	-4.141326	1.15E-101
PMI39_00399	6-phospho-beta-glucosidase	-3.768844	3.04E-94
PMI39_00400	PTS system, beta-glucosides-specific IIC component	-4.473577	2.84E-78
PMI39_00401	transcriptional regulator, RpiR family	-4.158164	6.35E-174
PMI39_00402	glutathione peroxidase	-3.902069	7.89E-67
PMI39_00403	transcriptional regulator, LacI family	-4.149601	6.52E-107
PMI39_00404	NAD(P)-dependent dehydrogenase, short-chain alcohol dehydrogenase family	-4.301434	1.19E-208
PMI39_00405	Ectoine hydroxylase-related dioxygenase, phytanoyl-CoA dioxygenase (PhyH) family	-4.340428	4.44E-269
PMI39_00406	Predicted arabinose efflux permease, MFS family	-4.358983	5.01E-175
PMI39_00407	hypothetical protein	-4.355022	3.38E-132
PMI39_00408	transcriptional regulator, LysR family	-4.430966	3.79E-271
PMI39_00409	Glycosyltransferase involved in cell wall bisynthesis	-4.350227	4.29E-150
PMI39_00411	Pimeloyl-ACP methyl ester carboxylesterase	-4.250622	1.09E-123
PMI39_00412	Predicted arabinose efflux permease, MFS family	-4.691445	1.05E-166
PMI39_00413	transcriptional regulator, AraC family	-4.555403	1.29E-115
PMI39_00414	Protein of unknown function (DUF1471)	-5.673713	1.40E-233
PMI39_00415	Protein of unknown function (DUF1203)	-4.425425	2.87E-118
PMI39_00416	Acetyltransferases	-4.176464	7.60E-192
PMI39_00417	Ribosomal protein S18 acetylase RimI	-4.098378	8.43E-143
PMI39_00418	Threonine/homoserine/homoserine lactone efflux protein	-4.128176	1.26E-144
PMI39_00422	hypothetical protein	-4.42343	1.51E-174
PMI39_00423	Nucleoside-diphosphate-sugar epimerase	-4.260684	8.57E-151
PMI39_00424	DNA-binding transcriptional regulator, LysR family	-4.002754	2.13E-257
PMI39_00425	hypothetical protein	-3.564994	4.18E-94
PMI39_00426	Pimeloyl-ACP methyl ester carboxylesterase	-3.738777	1.27E-105
PMI39_00427	DNA-binding response regulator, OmpR family, contains REC and winged-helix (wHTH) domain	-3.566039	9.10E-86

PMI39_00428	Signal transduction histidine kinase	-3.63855	7.54E-130
PMI39_00429	Pimeloyl-ACP methyl ester carboxylesterase	-3.709593	1.33E-67
PMI39_00430	Cytochrome c biogenesis protein CcdA	-3.746406	1.50E-220
PMI39_00431	aldehyde dehydrogenase (NAD+)	-3.880339	2.13E-144
PMI39_00432	DNA-binding transcriptional regulator, LysR family	-4.127299	1.59E-217
PMI39_00433	putative restriction endonuclease	-4.131317	1.13E-171
PMI39_00435	hypothetical protein	-4.21354	2.13E-106
PMI39_00437	hypothetical protein	-4.330222	1.34E-174
PMI39_00438	hypothetical protein	-4.20472	1.11E-77
PMI39_00454	Pimeloyl-ACP methyl ester carboxylesterase	-3.951701	3.92E-272
PMI39_00896	flagellar protein FlgJ	-6.089472	3.72E-161
PMI39_00935	Protoporphyrinogen oxidase	-3.62606	2.51E-171
PMI39_00937	hypothetical protein	-3.785523	7.02E-86
PMI39_00938	2-methylcitrate dehydratase PrpD	-3.582952	1.92E-182
PMI39_00939	amidohydrolase	-3.442435	1.24E-126
PMI39_00940	Acetyltransferase (GNAT) domain-containing protein	-3.455454	1.09E-93
PMI39_01724	formate dehydrogenase subunit gamma	-2.311072	5.41E-72
PMI39_02311	5-hydroxyisourate hydrolase	-3.608569	1.34E-125
PMI39_02312	AraC family transcriptional regulator, transcriptional activator FtrA	-3.901868	1.33E-168
PMI39_02313	drug resistance transporter, EmrB/QacA subfamily	-3.783325	8.93E-217
PMI39_02314	Pyridoxamine-phosphate oxidase	-3.76601	9.36E-79
PMI39_02315	transcriptional regulator, TetR family	-4.906643	1.54E-09
PMI39_02316	NAD(P)H dehydrogenase (quinone)	-3.879814	6.64E-75
PMI39_02317	outer membrane autotransporter barrel domain-containing protein	-3.958402	0
PMI39_02318	Uncharacterized membrane protein YfcC, ion transporter superfamily	-4.423153	2.97E-306
PMI39_02319	glutamate carboxypeptidase	-4.332136	3.34E-285
PMI39_02320	DNA-binding transcriptional regulator, LysR family	-4.563052	3.42E-251
PMI39_02322	hypothetical protein	-4.175121	3.29E-163
PMI39_02323	Pre-toxin domain with VENN motif-containing protein	-4.542985	2.06E-161
PMI39_02324	hypothetical protein	-4.832863	2.69E-170
PMI39_02325	hypothetical protein	-4.288155	1.98E-187
PMI39_02326	hypothetical protein	-4.470001	1.63E-132
PMI39_02327	Immunity protein 8	-4.481273	2.49E-288
PMI39_02328	Plasmid stabilization system protein ParE	-4.563714	2.19E-164
PMI39_02329	antitoxin ParD1/3/4	-4.507184	1.83E-256
PMI39_02330	hypothetical protein	-4.977299	1.23E-102
PMI39_02332	hypothetical protein	-4.19208	3.47E-148

PMI39_02503	LysR family transcriptional regulator, regulator of gene expression of beta-lactamase	-3.825383	2.90E-111
PMI39_02504	beta-lactamase class C	-3.772125	1.83E-43
PMI39_02506	Predicted arabinose efflux permease, MFS family	-3.859712	1.76E-183
PMI39_02507	transcriptional regulator, TetR family	-4.097635	1.32E-83
PMI39_02508	two component transcriptional regulator, LuxR family	-3.970934	1.77E-292
PMI39_02509	HPt (histidine-containing phosphotransfer) domain-containing protein	-4.18935	1.48E-127
PMI39_02510	major type 1 subunit fimbrin (pilin)	-4.239955	2.06E-40
PMI39_02511	chaperone protein EcpD	-4.631201	1.08E-81
PMI39_02512	outer membrane usher protein	-4.548347	1.24E-63
PMI39_02513	Pilin (type 1 fimbria component protein)	-4.491306	2.16E-135
PMI39_02514	two-component system, NarL family, response regulator EvgA	-4.203675	1.26E-227
PMI39_02515	two-component system, NarL family, sensor histidine kinase EvgS	-4.364231	1.31E-183
PMI39_02516	two component transcriptional regulator, LuxR family	-3.84771	2.34E-120
PMI39_02517	two-component system, NarL family, sensor histidine kinase EvgS	-4.015152	1.17E-238
PMI39_02518	two component transcriptional regulator, LuxR family	-4.089643	3.95E-110
PMI39_02519	transcriptional regulator, RpiR family	-4.063905	7.61E-78
PMI39_02520	PTS system, cellobiose-specific IIB component	-3.726106	1.13E-57
PMI39_02521	PTS system, cellobiose-specific IIA component	-3.884395	5.39E-26
PMI39_02522	6-phospho-beta-glucosidase	-3.978147	7.47E-92
PMI39_02523	PTS system, cellobiose-specific IIC component	-4.367688	9.81E-196
PMI39_02524	Biofilm development protein YmgB/AriR	-4.097885	1.45E-62
PMI39_02526	L-arabinose isomerase	-4.026816	2.14E-146
PMI39_02527	Cytosine/adenosine deaminases	-4.130414	2.81E-186
PMI39_02528	Predicted oxidoreductase	-4.087784	1.68E-222
PMI39_02529	hypothetical protein	-3.474463	2.92E-56
PMI39_02530	molybdenum cofactor cytidylyltransferase	-4.00853	2.23E-172
PMI39_02531	xanthine dehydrogenase accessory factor	-3.828596	1.98E-158
PMI39_02532	xanthine dehydrogenase YagR molybdenum-binding subunit	-3.844601	0
PMI39_02533	xanthine dehydrogenase YagS FAD-binding subunit	-3.794436	6.54E-118
PMI39_02534	xanthine dehydrogenase YagT iron-sulfur-binding subunit	-4.317017	9.95E-196
PMI39_02535	serine/threonine-protein kinase HipA	-4.417617	3.48E-251
PMI39_02536	Helix-turn-helix	-4.324455	1.06E-190
PMI39_02537	DNA-binding transcriptional regulator, LysR family	-4.483514	4.87E-197
PMI39_02538	Protein of unknown function (DUF2798)	-3.489834	7.54E-43
PMI39_02540	ADP-ribose pyrophosphatase	-4.023634	1.26E-89
PMI39_02541	Transposase	-4.044908	7.53E-139
PMI39_02542	Catechol 2,3-dioxygenase	-3.681631	3.63E-124

PMI39_02543	Protein N-acetyltransferase, RimJ/RimL family	-5.654301	7.31E-07
PMI39_02544	DNA-binding transcriptional regulator, GntR family	-3.296332	2.64E-61
PMI39_02545	Cytosine/adenosine deaminase	-4.127303	8.78E-131
PMI39_02546	Cytosine/adenosine deaminase	-4.219282	1.96E-196
PMI39_02547	cytosine deaminase	-4.395187	2.12E-47
PMI39_02548	peptide/nickel transport system ATP-binding protein	-4.156784	3.17E-103
PMI39_02549	peptide/nickel transport system ATP-binding protein	-4.323801	8.89E-09
PMI39_02550	peptide/nickel transport system permease protein	-3.611322	3.58E-53
PMI39_02551	peptide/nickel transport system permease protein	-5.192735	1.15E-57
PMI39_02552	peptide/nickel transport system substrate-binding protein	-4.887684	9.76E-75
PMI39_02553	hypothetical protein	-5.142805	1.18E-98
PMI39_02554	hypothetical protein	-5.081735	4.23E-18
PMI39_02555	regulatory protein, luxR family	-4.458574	3.90E-54
PMI39_02556	two-component system, NarL family, sensor histidine kinase EvgS	-4.474027	1.04E-142
PMI39_02557	two component transcriptional regulator, LuxR family	-4.63091	2.77E-158
PMI39_02560	xylose isomerase	-4.542976	2.53E-93
PMI39_02561	lactoylglutathione lyase	-4.364759	3.69E-90
PMI39_02562	Sugar phosphate permease	-4.705583	1.51E-159
PMI39_02563	transcriptional regulator, DeoR family	-4.494722	2.11E-111
PMI39_02564	transcriptional regulator, LacI family	-4.386029	1.07E-216
PMI39_02565	monosaccharide ABC transporter substrate-binding protein, CUT2 family	-4.066917	6.44E-118
PMI39_02566	creatinine amidohydrolase	-4.397118	3.68E-64
PMI39_02567	DNA-binding transcriptional regulator, LysR family	-4.37304	5.71E-139
PMI39_02568	basic membrane protein A	-4.491495	2.14E-113
PMI39_02569	nucleoside ABC transporter ATP-binding protein	-3.624479	4.19E-95
PMI39_02570	simple sugar transport system permease protein	-3.550631	2.81E-75
PMI39_02571	simple sugar transport system permease protein	-3.942565	1.31E-77
PMI39_02572	Cupin domain-containing protein	-4.73815	2.58E-64
PMI39_02573	Nicotinamidase-related amidase	-3.77516	1.12E-61
PMI39_02574	5-methylthioadenosine/S-adenosylhomocysteine deaminase	-4.314484	2.87E-93
PMI39_02576	serine/threonine-protein kinase HipA	-4.272788	3.35E-188
PMI39_02577	2,4-dienoyl-CoA reductase	-3.773643	5.40E-121
PMI39_02578	Choline dehydrogenase	-4.00219	1.89E-243
PMI39_02579	Membrane bound FAD containing D-sorbitol dehydrogenase	-4.111215	2.04E-148
PMI39_02580	Cytochrome c, mono- and diheme variants	-4.217008	7.18E-112
PMI39_02581	hypothetical protein	-4.124113	6.03E-121
PMI39_02582	6-phosphogluconolactonase	-4.10822	1.92E-107

PMI39_02583	Ribulose-5-phosphate 4-epimerase/Fuculose-1-phosphate aldolase	-4.599227	4.12E-58
PMI39_02584	D-galactonate transporter	-4.626821	7.10E-86
PMI39_02585	transcriptional regulator, LacI family	-3.926047	2.68E-112
PMI39_02586	molybdate transport system substrate-binding protein	-3.679141	4.51E-70
PMI39_02587	L-lactate dehydrogenase (cytochrome)	-4.394261	9.26E-116
PMI39_02588	hypothetical protein	-4.511598	4.37E-52
PMI39_02589	Uncharacterized conserved protein YgbK, DUF1537 family	-4.479486	2.65E-74
PMI39_02590	tryptophan-specific transport protein	-4.64857	1.09E-93
PMI39_02591	myo-inositol 2-dehydrogenase / D-chiro-inositol 1-dehydrogenase	-4.274503	1.94E-97
PMI39_02592	2-keto-myo-inositol isomerase	-4.060995	1.50E-137
PMI39_02593	MFS transporter, SP family, major inositol transporter	-3.784518	1.30E-149
PMI39_02594	hypothetical protein	-3.791728	1.56E-69
PMI39_02595	methyl-accepting chemotaxis protein-2, aspartate sensor receptor	-4.2412	8.10E-95
PMI39_02596	methionyl aminopeptidase	-3.91715	6.09E-145
PMI39_02597	ParD-like antitoxin of type II toxin-antitoxin system	-3.564727	8.95E-52
PMI39_02599	hypothetical protein	-3.779062	9.80E-65
PMI39_02600	MFS transporter, sugar porter (SP) family	-4.346216	5.54E-83
PMI39_02601	Uncharacterized membrane protein YoaK, UPF0700 family	-4.400765	2.62E-110
PMI39_02602	PTS system, beta-glucosides-specific IIC component	-3.55847	4.96E-109
PMI39_02603	6-phospho-beta-glucosidase	-2.354012	1.57E-47
PMI39_02604	transcriptional antiterminator, BglG family	-4.205992	2.77E-154
PMI39_02605	flagellar hook-associated protein 2	-4.314456	2.74E-233
PMI39_02606	HNH endonuclease	-4.126638	6.13E-168
PMI39_02607	Glucosyl transferase GtrII	-4.378743	2.17E-196
PMI39_02609	5-methyltetrahydropteroyltriglutamate--homocysteine methyltransferase	-3.971038	5.33E-187
PMI39_02610	protein of unknown function (DUF1852)	-3.656761	9.26E-171
PMI39_02612	hypothetical protein	-3.846546	3.76E-110
PMI39_02613	tRNA pseudouridine32 synthase / 23S rRNA pseudouridine746 synthase	-4.161592	2.14E-155
PMI39_02614	Cold shock protein, CspA family	-4.103545	1.38E-176
PMI39_02616	Protein of unknown function (DUF1493)	-3.447292	8.21E-37
PMI39_02617	hypothetical protein	-4.117243	1.10E-75
PMI39_02618	methyl-accepting chemotaxis protein	-4.160321	2.62E-273
PMI39_02620	glycogen operon protein	-3.768781	2.40E-223
PMI39_02621	(1->4)-alpha-D-glucan 1-alpha-D-glucosylmutase	-3.928274	3.96E-197
PMI39_02622	malto-oligosyltrehalose trehalohydrolase	-4.205862	8.77E-224
PMI39_02623	MFS transporter, MHS family, proline/betaine transporter	-4.408989	3.60E-147

PMI39_02624	hypothetical protein	-3.951048	2.91E-52
PMI39_02833	DNA-binding transcriptional regulator, LysR family	-4.127501	4.41E-101
PMI39_02834	aminobenzoyl-glutamate utilization protein B	-3.865156	3.52E-138
PMI39_02835	peptide/nickel transport system substrate-binding protein	-4.25528	2.41E-174
PMI39_02836	peptide/nickel transport system permease protein	-4.04083	1.23E-100
PMI39_02837	peptide/nickel transport system permease protein	-3.942933	6.74E-57
PMI39_02838	peptide/nickel transport system ATP-binding protein	-4.060985	2.06E-36
PMI39_02839	peptide/nickel transport system ATP-binding protein	-3.884901	1.63E-56
PMI39_02840	2-methylcitrate dehydratase PrpD	-4.192058	2.53E-136
PMI39_02841	NADP-dependent 3-hydroxy acid dehydrogenase YdfG	-3.933489	6.49E-132
PMI39_02842	hypothetical protein	-3.871195	2.17E-84
PMI39_02843	RNA polymerase sigma factor, sigma-70 family	-3.896	2.02E-158
PMI39_02844	L-ascorbate metabolism protein UlaG, beta-lactamase superfamily	-4.16442	1.23E-147
PMI39_02845	Uncharacterized conserved protein YtfP, gamma-glutamylcyclotransferase (GGCT)/AIG2-like family	-4.341642	1.07E-110
PMI39_02846	Uncharacterized conserved protein, DUF1778 family	-4.199961	5.92E-143
PMI39_02847	Acetyltransferase (GNAT) domain-containing protein	-4.389089	2.62E-138
PMI39_02848	hypothetical protein	-4.357277	4.17E-204
PMI39_02849	D-methionine transport system substrate-binding protein	-4.040125	1.15E-287
PMI39_02850	KUP system potassium uptake protein	-4.153928	4.09E-196
PMI39_02851	Protein of unknown function (DUF1471)	-4.462203	2.10E-177
PMI39_02852	DNA-binding transcriptional regulator, LysR family	-4.281474	2.39E-169
PMI39_02853	haloacetate dehalogenase	-4.237584	5.61E-100
PMI39_02854	3-isopropylmalate/(R)-2-methylmalate dehydratase small subunit	-4.314831	3.83E-91
PMI39_02855	3-isopropylmalate/(R)-2-methylmalate dehydratase large subunit	-4.386672	2.12E-76
PMI39_02856	malate dehydrogenase (quinone)	-3.938279	5.40E-237
PMI39_02857	L-lactate dehydrogenase (cytochrome)	-3.655438	1.10E-13
PMI39_02858	putative flavoprotein involved in K+ transport	-4.666621	1.75E-246
PMI39_02859	putative spermidine/putrescine transport system permease protein	-4.756132	5.65E-162
PMI39_02860	putative spermidine/putrescine transport system permease protein	-4.137429	1.34E-44
PMI39_02861	putative spermidine/putrescine transport system substrate-binding protein	-4.588801	7.36E-97
PMI39_02862	DNA-binding transcriptional regulator, LysR family	-4.601019	1.26E-71
PMI39_02863	glyoxylate/hydroxypyruvate reductase A	-4.327247	3.38E-132
PMI39_02864	3-dehydroquinate dehydratase	-3.777109	1.91E-56
PMI39_02865	glutathione S-transferase	-4.036539	5.53E-67
PMI39_02866	putative NAD(P)H quinone oxidoreductase, PIG3 family	-4.029122	5.77E-86

PMI39_02867	putative spermidine/putrescine transport system ATP-binding protein	-3.998453	1.12E-113
PMI39_02868	acetylornithine deacetylase	-3.935361	2.21E-77
PMI39_02869	Uncharacterized conserved protein, Ntn-hydrolase superfamily	-4.314782	2.34E-51
PMI39_02870	Enamine deaminase RidA, house cleaning of reactive enamine intermediates, YjgF/YER057c/UK114 family	-4.15731	5.15E-78
PMI39_02871	acyl-CoA thioester hydrolase	-3.979896	1.44E-57
PMI39_02872	Dienelactone hydrolase	-3.917833	3.95E-87
PMI39_02873	Sulfate permease and related transporters (MFS superfamily)	-4.653801	1.95E-139
PMI39_02875	Cd2+/Zn2+-exporting ATPase	-4.533804	4.73E-116
PMI39_02876	Protein of unknown function (DUF1471)	-4.456744	2.67E-116
PMI39_02877	gluconate:H+ symporter, GntP family	-4.240306	3.38E-181
PMI39_02878	Peptidoglycan/LPS O-acetylase OafA/YrhL, contains acyltransferase and SGNH-hydrolase domains	-4.343427	2.91E-163
PMI39_02879	transcriptional regulator, LacI family	-4.165394	2.12E-90
PMI39_02880	PTS system, arbutin-, cellobiose-, and salicin-specific IIC component	-4.087432	3.99E-105
PMI39_02881	6-phospho-beta-glucosidase	-3.90152	5.60E-174
PMI39_02882	Uncharacterized membrane protein YccC	-4.252981	2.80E-97
PMI39_02883	protein of unknown function (DUF4154)	-4.007141	1.30E-135
PMI39_02884	diguanylate cyclase (GGDEF) domain-containing protein	-3.963473	7.19E-222
PMI39_02885	Outer membrane protein and related peptidoglycan-associated (lipo)proteins	-4.238638	4.24E-173
PMI39_02886	UV-damage endonuclease	-3.878389	5.06E-188
PMI39_02887	choline/glycine/proline betaine transport protein	-3.768844	2.48E-87
PMI39_02888	transcriptional regulator, TetR family	-3.470398	4.78E-86
PMI39_02889	betaine aldehyde dehydrogenase	-3.292245	1.79E-131
PMI39_02890	choline dehydrogenase	-3.272344	5.60E-160
PMI39_02891	antitoxin VapB	-3.706984	4.73E-93
PMI39_02892	Acetyl esterase/lipase	-3.376526	1.02E-98
PMI39_02893	Nicotinamidase-related amidase	-3.654156	3.88E-82
PMI39_02894	cytosine permease	-3.947764	1.05E-07
PMI39_02895	aspartyl-tRNA(Asn)/glutamyl-tRNA(Gln) amidotransferase subunit A	-3.408261	1.51E-135
PMI39_02896	DNA-binding transcriptional regulator, LysR family	-3.287451	9.40E-61
PMI39_02897	Nucleoside-diphosphate-sugar epimerase	-3.822387	2.78E-107
PMI39_02898	small multidrug resistance pump	-4.392567	7.09E-111
PMI39_02899	Uncharacterized membrane protein YkgB	-4.18933	1.28E-121
PMI39_02900	Protein of unknown function (DUF1471)	-3.842353	3.44E-69
PMI39_02901	transcriptional regulator, TetR family	-4.210559	6.72E-241

PMI39_02902	iron complex transport system substrate-binding protein	-4.04613	3.95E-231
PMI39_02903	molybdate transport system substrate-binding protein	-3.808704	1.79E-159
PMI39_02904	Uncharacterized membrane protein HdeD, DUF308 family	-3.764457	4.85E-165
PMI39_02905	hypothetical protein	-4.438954	5.01E-118
PMI39_03108	L-lactate dehydrogenase (cytochrome)	-3.786932	2.00E-71
PMI39_03109	undecaprenyl phosphate-alpha-L-ara4N flippase subunit ArnF	-3.630142	4.35E-100
PMI39_03110	undecaprenyl phosphate-alpha-L-ara4N flippase subunit ArnE	-3.935862	7.02E-84
PMI39_03111	4-amino-4-deoxy-L-arabinose transferase	-3.47939	2.15E-176
PMI39_03112	undecaprenyl phosphate-alpha-L-ara4FN deformylase	-3.477436	2.29E-175
PMI39_03113	UDP-4-amino-4-deoxy-L-arabinose formyltransferase / UDP-glucuronic acid dehydrogenase (UDP-4-keto-hexauronic acid decarboxylating)	-3.556941	9.00E-152
PMI39_03114	undecaprenyl-phosphate 4-deoxy-4-formamido-L-arabinose transferase	-3.437698	5.21E-183
PMI39_03115	UDP-4-amino-4-deoxy-L-arabinose-oxoglutarate aminotransferase	-3.426194	2.69E-131
PMI39_03116	hypothetical protein	-4.515944	1.19E-96
PMI39_03117	leader peptidase (prepilin peptidase) / N-methyltransferase	-4.413205	1.23E-306
PMI39_03118	diaminobutyrate aminotransferase apoenzyme	-4.068895	2.60E-287
PMI39_03119	L-2,4-diaminobutyrate decarboxylase	-3.950543	2.14E-261
PMI39_03120	fibronectin-binding autotransporter adhesin	-4.131957	2.08E-137
PMI39_03121	Small-conductance mechanosensitive channel	-4.39246	7.36E-211
PMI39_03122	Phosphoserine phosphatase	-4.144906	2.02E-288
PMI39_03123	Oxygen tolerance	-4.111139	1.09E-108
PMI39_03124	Ca-activated chloride channel family protein	-3.823446	1.49E-157
PMI39_03125	Ca-activated chloride channel family protein	-3.920405	6.67E-77
PMI39_03126	protein of unknown function (DUF4381)	-3.450258	1.10E-45
PMI39_03127	Uncharacterized conserved protein (some members contain a von Willebrand factor type A (vWA) domain)	-2.394857	0.001108283
PMI39_03128	MoxR-like ATPase	-3.26715	0.004580315
PMI39_03129	uncharacterized protein	-4.083276	1.74E-206
PMI39_03132	long-chain fatty acid transport protein	-4.187548	5.85E-181
PMI39_03133	hypothetical protein	-4.041534	1.94E-126
PMI39_03134	diguanylate cyclase (GGDEF) domain-containing protein	-4.092872	2.21E-196
PMI39_03135	Purine-cytosine permease	-4.716136	3.08E-183
PMI39_03136	Predicted amidohydrolase	-4.373742	9.87E-179
PMI39_03137	amino acid/amide ABC transporter substrate-binding protein, HAAT family	-3.842091	1.04E-155
PMI39_03138	Two-component response regulator, AmiR/NasT family, consists of REC and RNA-binding antiterminator (ANTAR) domains	-3.655088	6.62E-68
PMI39_03139	DNA-binding transcriptional regulator, LysR family	-3.984852	1.49E-76

PMI39_03140	Predicted oxidoreductase	-3.738118	1.02E-124
PMI39_03141	GntR family transcriptional regulator / MocR family aminotransferase	-4.021411	1.34E-94
PMI39_03142	4-aminobutyrate aminotransferase	-3.138018	1.88E-101
PMI39_03143	GABA permease	-3.737252	6.04E-132
PMI39_03144	hypothetical protein	-3.432319	5.20E-56
PMI39_03145	Uncharacterized lipoprotein YddW, UPF0748 family	-3.852785	1.03E-194
PMI39_03387	molybdate/tungstate transport system substrate-binding protein	-4.119089	1.80E-79
PMI39_03388	hypothetical protein	-4.317568	2.66E-110
PMI39_03389	hypothetical protein	-3.917198	1.06E-83
PMI39_03390	uncharacterized zinc-type alcohol dehydrogenase-like protein	-4.072406	4.23E-133
PMI39_03391	uncharacterized zinc-type alcohol dehydrogenase-like protein	-3.816596	1.54E-102
PMI39_03392	Uncharacterized iron-regulated membrane protein	-3.885416	5.39E-113
PMI39_03393	Protein of unknown function (DUF2946)	-3.554653	4.90E-71
PMI39_03394	L-glutaminase	-4.179564	3.23E-205
PMI39_03395	cupin 2 domain-containing protein	-4.253383	7.46E-90
PMI39_03396	Uncharacterized protein, UPF0303 family	-4.271541	5.22E-125
PMI39_03397	Cytochrome c, mono- and diheme variants	-3.819224	4.70E-116
PMI39_03398	gluconate 2-dehydrogenase alpha chain	-4.386262	3.89E-198
PMI39_03399	gluconate 2-dehydrogenase gamma chain	-3.81056	3.80E-57
PMI39_03400	Sugar phosphate isomerase/epimerase	-3.921146	3.16E-127
PMI39_03402	Predicted dehydrogenase	-3.658191	7.79E-133
PMI39_03403	Sugar phosphate isomerase/epimerase	-3.864349	4.96E-84
PMI39_03404	DNA-binding transcriptional regulator, ArsR family	-3.883843	9.18E-146
PMI39_03405	MFS transporter, CP family, cyanate transporter	-3.513165	2.82E-89
PMI39_03406	RHH-type transcriptional regulator, rel operon repressor / antitoxin RelB	-4.13278	2.60E-102
PMI39_03407	beta-carotene 3-hydroxylase	-3.969177	1.33E-219
PMI39_03408	phytoene synthase	-3.81807	8.54E-101
PMI39_03409	phytoene desaturase	-3.679885	2.52E-287
PMI39_03410	lycopene beta-cyclase	-2.358535	4.23E-09
PMI39_03411	UDP-glucoronosyl and UDP-glucosyl transferase	-3.530769	4.79E-114
PMI39_03412	geranylgeranyl diphosphate synthase, type II	-3.94146	2.75E-122
PMI39_03413	hydroxypyruvate reductase	-3.988826	2.33E-109
PMI39_03414	Glycine/D-amino acid oxidase (deaminating)	-3.89107	1.43E-221
PMI39_03415	DNA-binding transcriptional regulator, MocR family, contains an aminotransferase domain	-4.504798	2.61E-227
PMI39_03416	EamA-like transporter family protein	-4.511233	1.00E-157
PMI39_03417	trehalose 6-phosphate synthase	-4.524627	2.97E-127

PMI39_03418	Predicted dehydrogenase	-4.115624	3.92E-164
PMI39_03419	Ribosomal protein S18 acetylase RimI	-4.267839	1.62E-285
PMI39_03420	Phage antirepressor protein YoqD, KilAC domain	-4.207824	0
PMI39_03421	Mannosyltransferase OCH1 and related enzymes	-4.402621	4.73E-169
PMI39_03422	Helix-turn-helix domain-containing protein	-3.967826	2.76E-255
PMI39_03423	Arabinose efflux permease	-4.142532	1.33E-173
PMI39_03424	Beta-barrel assembly machine subunit BamA	-4.430123	2.83E-120
PMI39_03425	multiple sugar transport system ATP-binding protein	-4.052948	5.23E-82
PMI39_03426	Sugar or nucleoside kinase, ribokinase family	-4.856409	1.86E-10
PMI39_03427	multiple sugar transport system permease protein	-3.850898	6.96E-69
PMI39_03428	multiple sugar transport system permease protein	-4.540708	3.43E-112
PMI39_03429	multiple sugar transport system substrate-binding protein	-4.217955	8.73E-109
PMI39_03430	hypothetical protein	-4.054245	1.14E-101
PMI39_03431	hypothetical protein	-3.982597	3.66E-85
PMI39_03432	hypothetical protein	-4.23945	1.31E-142
PMI39_03433	Sugar kinase of the NBD/HSP70 family, may contain an N-terminal HTH domain	-3.899353	2.86E-221
PMI39_03434	L-asparagine transporter	-4.406581	3.53E-141
PMI39_03435	glutamate dehydrogenase (NAD(P)+)	-4.841749	1.18E-198
PMI39_03436	hypothetical protein	-4.086055	7.98E-80
PMI39_03437	hypothetical protein	-3.881512	1.43E-102
PMI39_03438	DNA-binding transcriptional regulator, LysR family	-4.046808	3.28E-103
PMI39_03440	Phenylpyruvate tautomerase PptA, 4-oxalocrotonate tautomerase family	-3.895305	1.70E-40
PMI39_03441	glutathione transport system ATP-binding protein	-3.666877	8.04E-165
PMI39_03442	Microcystin degradation protein MlrC, contains DUF1485 domain	-3.880305	3.19E-152
PMI39_03443	Sugar kinase of the NBD/HSP70 family, may contain an N-terminal HTH domain	-4.029575	1.75E-161
PMI39_03444	glutathione transport system substrate-binding protein	-3.93433	6.71E-89
PMI39_03445	glutathione transport system permease protein	-3.744537	2.17E-121
PMI39_03446	glutathione transport system permease protein	-4.046998	1.93E-66
PMI39_03447	N-acetylglucosamine kinase	-4.263532	3.82E-09
PMI39_03628	hypothetical protein	-8.065918	4.02E-262
PMI39_03841	FAD/FMN-containing dehydrogenase	-4.368813	3.02E-218
PMI39_03842	amylovoran biosynthesis protein AmsF	-4.553305	1.72E-250
PMI39_03843	outer membrane lipase/esterase	-4.301921	2.65E-212
PMI39_03844	methyl-accepting chemotaxis sensory transducer with Pas/Pac sensor	-4.096119	3.16E-143
PMI39_03845	Glycine/D-amino acid oxidase (deaminating)	-4.305288	0

PMI39_03846	transcriptional regulator, XRE family with cupin sensor	-4.292899	1.15E-129
PMI39_03847	polar amino acid transport system substrate-binding protein	-4.209338	2.93E-64
PMI39_03848	polar amino acid transport system permease protein	-4.117346	9.96E-125
PMI39_03849	polar amino acid transport system ATP-binding protein	-4.137762	1.59E-152
PMI39_03850	hypothetical protein	-4.048844	2.78E-97
PMI39_03851	manganese/iron transport system substrate-binding protein	-4.806525	6.49E-276
PMI39_03852	manganese/iron transport system ATP-binding protein	-4.721495	1.31E-207
PMI39_03853	manganese/iron transport system permease protein	-4.542307	3.30E-228
PMI39_03854	manganese/iron transport system permease protein	-4.547951	1.27E-170
PMI39_03855	Enamine deaminase RidA, house cleaning of reactive enamine intermediates, YjgF/YER057c/UK114 family	-4.225228	1.15E-121
PMI39_03856	flavin reductase	-4.175568	2.33E-82
PMI39_03857	Predicted oxidoreductase	-4.119708	1.22E-100
PMI39_03858	aminoacrylate peracid reductase	-4.183826	7.76E-83
PMI39_03859	simple sugar transport system permease protein	-4.371559	7.75E-79
PMI39_03860	simple sugar transport system permease protein	-4.388384	4.39E-104
PMI39_03861	simple sugar transport system ATP-binding protein	-4.024232	9.64E-101
PMI39_03862	basic membrane protein A	-4.436655	1.28E-116
PMI39_03863	pyrimidine oxygenase	-3.867092	3.19E-94
PMI39_03864	aminoacrylate hydrolase	-3.809969	1.71E-65
PMI39_03865	DNA-binding transcriptional regulator, LysR family	-4.247004	5.69E-151
PMI39_03866	outer membrane autotransporter barrel domain-containing protein	-4.281824	4.48E-169
PMI39_03867	DNA-binding transcriptional regulator, Lrp family	-4.106043	3.88E-180
PMI39_03868	Protein of unknown function (DUF2000)	-4.238443	1.37E-171
PMI39_03869	AraC-type DNA-binding protein	-3.547881	7.07E-178
PMI39_03870	NADP-dependent 3-hydroxy acid dehydrogenase YdfG	-4.153713	4.25E-75
PMI39_03871	transcriptional regulator, XRE family with cupin sensor	-3.867424	4.32E-86
PMI39_03872	B3/B4 domain-containing protein (DNA/RNA-binding domain of Phe-tRNA-synthetase)	-4.130808	3.79E-71
PMI39_03873	Predicted N-acetyltransferase YhbS	-4.168469	2.20E-85
PMI39_03874	8-oxo-dGTP diphosphatase	-4.146509	1.47E-133
PMI39_03875	protein of unknown function (DUF1543)	-4.101062	2.44E-144
PMI39_03876	MFS transporter, CP family, cyanate transporter	-4.207416	1.77E-110
PMI39_03877	DNA-binding transcriptional regulator, MarR family	-3.988205	1.20E-130
PMI39_03878	cytochrome b561	-4.14085	1.31E-192
PMI39_03879	Transcriptional regulator containing an amidase domain and an AraC-type DNA-binding HTH domain	-3.88038	4.00E-115
PMI39_03880	Predicted O-methyltransferase	-3.780448	3.31E-106
PMI39_03881	Predicted DNA-binding protein, MmcQ/YjbR family	-3.767032	1.07E-74

PMI39_03882	hypothetical protein	-4.091127	4.04E-172
PMI39_03883	transcriptional regulator, TetR family	-4.451555	2.70E-122
PMI39_03884	Predicted arabinose efflux permease, MFS family	-4.782544	1.65E-69
PMI39_03885	Predicted HD superfamily hydrolase	-4.49008	1.01E-45
PMI39_03886	Transcriptional regulator GlxA family, contains an amidase domain and an AraC-type DNA-binding HTH domain	-3.685455	3.48E-136
PMI39_03887	non-heme chloroperoxidase	-3.882837	1.67E-119
PMI39_03888	Transcriptional regulator GlxA family, contains an amidase domain and an AraC-type DNA-binding HTH domain	-3.582048	2.82E-151
PMI39_03889	Predicted arabinose efflux permease, MFS family	-3.636081	6.58E-202
PMI39_03890	methyl-accepting chemotaxis sensory transducer with Cache sensor	-3.640931	2.46E-246
PMI39_03891	Fic family protein	-3.741595	6.08E-274
PMI39_03892	hypothetical protein	-3.679887	1.98E-73
PMI39_03893	SIR2-like domain-containing protein	-4.269512	4.33E-159
PMI39_03894	XTP/dITP diphosphohydrolase	-4.184382	8.65E-78
PMI39_03895	EAL domain, c-di-GMP-specific phosphodiesterase class I (or its enzymatically inactive variant)	-4.259413	4.63E-189
PMI39_03897	hypothetical protein	-4.004743	5.16E-111
PMI39_03898	polar amino acid transport system substrate-binding protein	-3.942371	3.29E-89
PMI39_03899	polar amino acid transport system ATP-binding protein	-3.688261	4.10E-113
PMI39_03900	polar amino acid transport system permease protein	-3.813245	1.35E-76
PMI39_03901	N-acetylglutamate synthase and related acetyltransferases	-3.785762	1.24E-66
PMI39_03902	polar amino acid transport system substrate-binding protein	-4.080552	2.90E-118
PMI39_03903	FMN-dependent oxidoreductase, nitrilotriacetate monooxygenase family	-3.679235	2.03E-123
PMI39_03904	amidohydrolase	-3.970055	7.49E-116
PMI39_03905	luciferase family oxidoreductase, group 1	-3.789618	2.39E-100
PMI39_03906	tripartite ATP-independent transporter solute receptor, DctP family	-4.085289	4.40E-227
PMI39_03907	TRAP-type C4-dicarboxylate transport system, small permease component	-3.929616	2.66E-119
PMI39_03908	TRAP transporter, DctM subunit	-4.379356	4.45E-140
PMI39_03909	peptide/nickel transport system substrate-binding protein	-3.480945	1.11E-54
PMI39_03910	peptide/nickel transport system permease protein	-3.026893	5.56E-33
PMI39_03911	peptide/nickel transport system permease protein	-3.183953	1.50E-88
PMI39_03912	peptide/nickel transport system ATP-binding protein	-3.380879	7.56E-125
PMI39_03913	putative FMN-dependent luciferase-like monooxygenase, KPN_01858 family	-3.190191	1.60E-96
PMI39_03914	alkylhydroperoxidase domain protein, Avi_7169 family	-3.025784	2.26E-110
PMI39_03915	amidohydrolase	-3.607044	1.83E-87

PMI39_03916	Glyoxalase-like domain-containing protein	-3.423026	1.20E-130
PMI39_03917	Protein N-acetyltransferase, RimJ/RimL family	-3.318488	6.23E-120
PMI39_03918	hypothetical protein	-3.613826	4.53E-206
PMI39_03919	4-hydroxy-tetrahydrodipicolinate synthase	-3.480037	2.80E-217
PMI39_03920	transcriptional regulator, GntR family	-3.621747	1.25E-108
PMI39_03921	leucine efflux protein	-3.94514	6.17E-115
PMI39_03922	quaternary ammonium compound-resistance protein SugE	-3.821436	5.68E-90
PMI39_03923	Predicted transcriptional regulators	-3.53777	2.12E-167
PMI39_03924	B3/B4 domain-containing protein (DNA/RNA-binding domain of Phe-tRNA-synthetase)	-3.431631	2.45E-104
PMI39_03925	L-lysine exporter family protein LysE/ArgO	-3.585573	3.55E-217
PMI39_03926	outer membrane autotransporter barrel domain-containing protein	-3.495599	4.78E-262
PMI39_03927	hypothetical protein	-4.362462	8.57E-129
PMI39_03928	hypothetical protein	-4.249075	6.59E-102
PMI39_03929	Outer membrane protein and related peptidoglycan-associated (lipo)proteins	-4.638159	6.32E-85
PMI39_03930	AraC-type DNA-binding protein	-4.549498	2.91E-164
PMI39_03931	2,4-dienoyl-CoA reductase	-4.387369	1.87E-191
PMI39_03932	Predicted oxidoreductase	-4.273177	7.87E-251
PMI39_03933	hypothetical protein	-4.170395	8.38E-56
PMI39_04053	2-keto-4-pentenoate hydratase/2-oxohepta-3-ene-1,7-dioic acid hydratase (catechol pathway)	-3.992186	5.90E-107
PMI39_04054	2-keto-3-deoxy-L-fuconate dehydrogenase	-3.914772	3.21E-119
PMI39_04055	DNA-binding transcriptional regulator, FadR family	-4.089338	4.78E-128
PMI39_04056	ABC transporter transmembrane region	-4.044201	3.65E-91
PMI39_04057	transcriptional regulator, TetR family	-4.27273	5.37E-200
PMI39_04058	Membrane protein involved in the export of O-antigen and teichoic acid	-4.643874	1.95E-108
PMI39_04059	TupA-like ATPgrasp	-4.304669	1.83E-114
PMI39_04060	Small-conductance mechanosensitive channel	-4.188	3.23E-119
PMI39_04061	O-acetylserine/cysteine efflux transporter	-4.416921	8.60E-79
PMI39_04062	aspartyl-tRNA(Asn)/glutamyl-tRNA(Gln) amidotransferase subunit A	-3.842805	5.03E-143
PMI39_04063	hypothetical protein	-4.313859	2.09E-34
PMI39_04064	NitT/TauT family transport system substrate-binding protein	-4.020822	1.97E-111
PMI39_04065	NitT/TauT family transport system permease protein	-4.518876	6.46E-77
PMI39_04066	NitT/TauT family transport system ATP-binding protein	-4.65424	6.64E-74
PMI39_04067	Predicted amidohydrolase	-3.732716	2.57E-72
PMI39_04068	transcriptional regulator, XRE family with cupin sensor	-4.255248	5.75E-102
PMI39_04069	Organic hydroperoxide reductase OsmC/OhrA	-3.840677	6.38E-158

PMI39_04070	diguanylate cyclase (GGDEF) domain-containing protein	-4.158017	1.77E-200
PMI39_04071	Methyl-accepting chemotaxis protein	-3.944733	0
PMI39_04072	DNA-binding transcriptional regulator, LysR family	-4.483603	0
PMI39_04073	Predicted ester cyclase	-4.305793	6.03E-99
PMI39_04074	uncharacterized zinc-type alcohol dehydrogenase-like protein	-4.324353	2.55E-101
PMI39_04075	transcriptional regulator, HxlR family	-3.862192	1.53E-58
PMI39_04076	Helix-turn-helix domain-containing protein	-4.143629	4.88E-115
PMI39_04078	Nucleoside-diphosphate-sugar epimerase	-4.323971	3.44E-136
PMI39_04079	hypothetical protein	-4.468902	6.32E-234
PMI39_04080	hypothetical protein	-4.702321	7.86E-116
PMI39_04081	Biotin carboxylase	-4.4255	5.67E-140
PMI39_04082	Permease of the drug/metabolite transporter (DMT) superfamily	-4.138994	4.57E-71
PMI39_04083	hypothetical protein	-4.542037	6.02E-172
PMI39_04084	transcriptional regulator, TetR family	-4.245582	3.14E-68
PMI39_04085	Predicted O-methyltransferase	-4.273949	7.27E-102
PMI39_04086	GTPase, G3E family	-4.68344	4.81E-87
PMI39_04087	iron complex transport system substrate-binding protein	-3.734481	8.69E-71
PMI39_04088	transcriptional regulator, IclR family	-4.121135	1.74E-155
PMI39_04089	Enamine deaminase RidA, house cleaning of reactive enamine intermediates, YjgF/YER057c/UK114 family	-4.295618	4.05E-88
PMI39_04090	polar amino acid transport system substrate-binding protein	-4.199638	2.69E-110
PMI39_04091	polar amino acid transport system permease protein	-4.315292	2.52E-119
PMI39_04092	polar amino acid transport system permease protein	-4.692621	3.39E-109
PMI39_04093	polar amino acid transport system ATP-binding protein	-4.470433	2.95E-81
PMI39_04094	D-serine deaminase, pyridoxal phosphate-dependent	-3.890052	2.94E-87
PMI39_04095	aspartate racemase	-4.444003	2.28E-136
PMI39_04096	Uncharacterized membrane protein YccC	-4.399164	9.68E-155
PMI39_04097	sulfonate transport system substrate-binding protein	-4.384859	1.71E-189
PMI39_04099	Acyl-CoA dehydrogenase	-4.162931	1.69E-124
PMI39_04100	Predicted arabinose efflux permease, MFS family	-4.243639	7.12E-126
PMI39_04101	ABC-type metal ion transport system, substrate-binding protein/surface antigen	-3.785379	1.39E-120
PMI39_04102	ABC-type methionine transport system, permease component	-3.756147	2.92E-110
PMI39_04103	D-methionine transport system ATP-binding protein	-3.934873	7.08E-65
PMI39_04104	FMN-dependent oxidoreductase, nitrilotriacetate monooxygenase family	-3.471582	2.46E-72
PMI39_04105	Uncharacterized NAD(P)/FAD-binding protein YdhS	-3.598759	5.43E-211
PMI39_04106	alkanesulfonate monooxygenase	-3.761671	8.30E-136

PMI39_04107	ABC transporter, substrate-binding protein, aliphatic sulfonates family	-4.29071	1.58E-91
PMI39_04108	chromosome partitioning protein, ParB family	-3.570124	2.08E-163
PMI39_04109	Cellulose biosynthesis protein BcsQ	-3.955752	5.70E-275
PMI39_04110	Initiator Replication protein	-3.982311	1.10E-127
PMI39_04111	succinate-semialdehyde dehydrogenase	-4.622004	3.27E-90
PMI39_04112	nucleoside transporter	-4.683156	3.49E-129
PMI39_04113	ADP-ribosylglycohydrolase	-3.960585	9.14E-154
PMI39_04114	Sugar or nucleoside kinase, ribokinase family	-3.89177	1.99E-135
PMI39_04115	DNA-binding transcriptional regulator, GntR family	-3.93443	3.94E-194
PMI39_04116	ADP-heptose:LPS heptosyltransferase	-3.530988	1.29E-49
PMI39_04117	4-oxalocrotonate tautomerase	-4.139767	2.23E-80
PMI39_04118	peptide/nickel transport system substrate-binding protein	-3.991721	6.80E-77
PMI39_04119	peptide/nickel transport system permease protein	-3.950737	5.04E-68
PMI39_04120	peptide/nickel transport system permease protein	-4.358257	2.47E-85
PMI39_04121	peptide/nickel transport system ATP-binding protein	-3.838854	3.19E-164
PMI39_04122	phosphinothricin acetyltransferase	-3.834934	2.14E-80
PMI39_04123	transcriptional regulator, XRE family with cupin sensor	-4.358384	2.09E-143
PMI39_04124	4-azaleucine resistance probable transporter AzlC	-3.836826	1.11E-112
PMI39_04125	Branched-chain amino acid transport protein (AzlD)	-3.775214	7.55E-85
PMI39_04126	Protein N-acetyltransferase, RimJ/RimL family	-3.488018	2.06E-97
PMI39_04127	N-acetylglutamate synthase and related acetyltransferases	-3.769334	7.37E-202
PMI39_04128	magnesium transporter	-3.90509	7.98E-161
PMI39_04129	cyclohexyl-isocyanide hydratase	-4.017067	2.48E-161
PMI39_04130	NADP-dependent 3-hydroxy acid dehydrogenase YdfG	-3.902584	3.60E-65
PMI39_04131	AraC-type DNA-binding protein	-3.653042	2.73E-123
PMI39_04133	NAD(P)-dependent dehydrogenase, short-chain alcohol dehydrogenase family	-4.019911	4.06E-68
PMI39_04134	LysR family transcriptional regulator, glycine cleavage system transcriptional activator	-3.479341	7.88E-124
PMI39_04135	amidohydrolase	-3.625233	4.86E-129
PMI39_04136	octopine/nopaline transport system substrate-binding protein	-3.928295	1.35E-121
PMI39_04137	octopine/nopaline transport system permease protein	-3.833842	2.91E-14
PMI39_04138	octopine/nopaline transport system permease protein	-3.542876	5.76E-103
PMI39_04139	octopine/nopaline transport system ATP-binding protein	-3.746512	4.27E-95
PMI39_04140	His Kinase A (phospho-acceptor) domain-containing protein	-3.817806	3.34E-145
PMI39_04141	two component transcriptional regulator, LuxR family	-3.608949	4.51E-121
PMI39_04142	Uncharacterized conserved protein GlcG, DUF336 family	-3.631351	1.01E-79
PMI39_04161	ArsR family transcriptional regulator	-4.442047	9.22E-198

PMI39_04162	arsenical pump membrane protein	-4.057941	4.14E-167
PMI39_04163	arsenate reductase	-4.066336	1.57E-83
PMI39_04164	arsenical resistance protein ArsH	-3.708813	1.41E-77
PMI39_04165	DNA-binding transcriptional regulator, ArsR family	-4.298366	3.79E-109
PMI39_04166	Phage integrase family protein	-4.404508	1.07E-137
PMI39_04167	Excisionase-like protein	-4.524499	3.17E-102
PMI39_04168	hypothetical protein	-4.848758	7.35E-113
PMI39_04169	hypothetical protein	-4.080309	1.23E-62
PMI39_04170	hypothetical protein	-4.474707	1.95E-111
PMI39_04172	hypothetical protein	-4.4863	5.40E-80
PMI39_04173	hypothetical protein	-4.203619	1.05E-106
PMI39_04174	hypothetical protein	-4.414262	3.50E-172
PMI39_04175	DNA polymerase-3 subunit theta	-4.222363	2.65E-91
PMI39_04176	Putative SOS response-associated peptidase YedK	-4.354785	2.02E-106
PMI39_04177	YadA-like C-terminal region	-4.443776	4.96E-88
PMI39_04179	hypothetical protein	-4.781786	1.80E-133
PMI39_04180	RecT family protein	-4.517296	3.02E-169
PMI39_04181	exodeoxyribonuclease VIII	-4.620318	5.31E-147
PMI39_04182	hypothetical protein	-4.526007	1.44E-122
PMI39_04183	hypothetical protein	-4.2401	1.83E-101
PMI39_04185	Uncharacterized low-complexity proteins	-4.279934	1.59E-105
PMI39_04187	hypothetical protein	-4.354018	8.24E-90
PMI39_04189	hypothetical protein	-4.750205	5.69E-91
PMI39_04190	SOS-response transcriptional repressor LexA (RecA-mediated autopeptidase)	-3.97271	1.45E-115
PMI39_04192	hypothetical protein	-5.039282	1.49E-107
PMI39_04204	D-methionine transport system permease protein	-4.306888	6.58E-68
PMI39_04205	D-methionine transport system ATP-binding protein	-3.858064	4.83E-115
PMI39_04206	D-methionine transport system substrate-binding protein	-4.256603	1.61E-100
PMI39_04207	FMN-dependent oxidoreductase, nitrilotriacetate monooxygenase family	-4.048002	3.71E-177
PMI39_04208	sulfur acquisition oxidoreductase, SfnB family	-3.692692	1.54E-71
PMI39_04209	sulfur acquisition oxidoreductase, SfnB family	-3.748211	1.05E-104
PMI39_04210	hypothetical protein	-3.981953	2.39E-242
PMI39_04211	amidase	-3.737833	5.91E-137
PMI39_04212	Transcriptional regulator containing an amidase domain and an AraC-type DNA-binding HTH domain	-3.680252	4.97E-79
PMI39_04213	Two-component response regulator, AmiR/NasT family, consists of REC and RNA-binding antiterminator (ANTAR) domains	-3.682044	8.94E-60

PMI39_04214	urea transport system ATP-binding protein	-3.680826	5.22E-161
PMI39_04215	urea transport system ATP-binding protein	-3.954993	4.84E-140
PMI39_04216	branched-chain amino acid transport system permease protein	-4.084927	2.48E-10
PMI39_04217	branched-chain amino acid transport system permease protein	-3.672408	4.73E-106
PMI39_04218	branched-chain amino acid transport system substrate-binding protein	-4.032005	2.29E-115
PMI39_04219	6-phosphogluconolactonase, cycloisomerase 2 family	-4.306265	5.06E-141
PMI39_04220	L-lysine exporter family protein LysE/ArgO	-3.752147	1.19E-76
PMI39_04221	myo-inositol-1(or 4)-monophosphatase	-3.730299	3.09E-52
PMI39_04222	iron(III) transport system ATP-binding protein	-4.446058	1.40E-06
PMI39_04223	iron(III) transport system permease protein	-3.996068	9.24E-123
PMI39_04224	iron(III) transport system substrate-binding protein	-4.081388	2.47E-70
PMI39_04225	transcriptional regulator, LacI family	-3.696768	1.11E-129
PMI39_04226	DNA-binding transcriptional regulator, LysR family	-3.545919	1.33E-128
PMI39_04227	NAD(P)-dependent dehydrogenase, short-chain alcohol dehydrogenase family	-3.661646	1.82E-52
PMI39_04228	DNA-binding transcriptional regulator, LysR family	-3.834321	9.09E-40
PMI39_04229	Zn-dependent hydrolases, including glyoxylases	-4.261769	2.64E-80
PMI39_04230	mandelamide amidase	-3.678782	3.31E-147
PMI39_04231	hypothetical protein	-3.908799	1.99E-98
PMI39_04232	AraC-type DNA-binding protein	-3.581187	3.08E-82
PMI39_04233	anion transporter	-3.875866	7.72E-173
PMI39_04273	FMN-dependent oxidoreductase, nitrilotriacetate monooxygenase family	-3.392289	3.97E-106
PMI39_04274	aspartate aminotransferase	-3.814414	4.16E-226
PMI39_04275	adenosylhomocysteine nucleosidase	-4.058092	5.93E-305
PMI39_04276	DNA-binding protein H-NS	-3.970277	1.61E-127
PMI39_04277	outer-membrane receptor for ferric coprogen and ferric-rhodotorulic acid	-4.261975	3.39E-221
PMI39_04278	succinyl-diaminopimelate desuccinylase	-3.608314	0.035026785
PMI39_04279	Uncharacterized membrane protein	-4.110017	4.64E-132
PMI39_04280	phosphotriesterase-related protein	-4.19359	2.48E-96
PMI39_04281	monosaccharide ABC transporter membrane protein, CUT2 family	-4.040382	5.83E-167
PMI39_04282	monosaccharide ABC transporter ATP-binding protein, CUT2 family	-4.854697	3.12E-66
PMI39_04283	transcriptional regulator, LacI family	-3.765895	4.31E-187
PMI39_04284	ribose transport system substrate-binding protein	-4.36757	1.05E-119
PMI39_04285	ribokinase	-4.140743	3.37E-96
PMI39_04286	hypothetical protein	-4.097175	7.67E-87
PMI39_04287	thiaminase (transcriptional activator TenA)	-4.342453	8.96E-92

PMI39_04288	ABC transporter	-4.00247	1.86E-105
PMI39_04289	rhamnose transport system substrate-binding protein	-4.436382	2.44E-97
PMI39_04290	AI-2 transport system permease protein	-4.625393	2.62E-85
PMI39_04291	ribose transport system permease protein	-3.937798	5.64E-96
PMI39_04292	acetylornithine deacetylase	-3.983993	8.16E-66
PMI39_04293	alkanesulfonate monooxygenase	-3.775942	1.17E-72
PMI39_04294	Acyl-CoA dehydrogenase	-4.13685	1.66E-84
PMI39_04295	NitT/TauT family transport system substrate-binding protein	-3.71799	3.45E-65
PMI39_04296	cysteine synthase A	-3.661079	4.02E-125
PMI39_04297	NitT/TauT family transport system permease protein	-3.748629	1.31E-85
PMI39_04299	NitT/TauT family transport system ATP-binding protein	-3.890688	5.25E-97
PMI39_04302	lipid A ethanolaminephosphotransferase	-4.290606	2.54E-274
PMI39_04303	Uncharacterized membrane-anchored protein	-4.507221	2.19E-98
PMI39_04304	two-component system, OmpR family, response regulator	-4.027094	2.54E-56
PMI39_04305	two-component system, OmpR family, sensor kinase	-4.168552	8.05E-113
PMI39_04306	undecaprenyl-diphosphatase	-4.253916	1.51E-124
PMI39_04307	phosphatidylglycerophosphatase B	-4.630789	1.40E-117
PMI39_04309	iron complex transport system substrate-binding protein	-4.413797	7.95E-50
PMI39_04310	iron complex outermembrane recepter protein	-4.240499	3.00E-119
PMI39_04311	transcriptional regulator, RpiR family	-4.291815	2.82E-99
PMI39_04312	putative ABC transport system ATP-binding protein	-3.65684	1.06E-76
PMI39_04313	putative ABC transport system permease protein	-3.657022	4.85E-72
PMI39_04314	transcriptional regulator, LysR family	-4.047628	3.17E-158
PMI39_04315	hypothetical protein	-4.195306	6.38E-130
PMI39_04316	2,4-dienoyl-CoA reductase	-3.659356	1.17E-227
PMI39_04350	MFS transporter, ENTS family, enterobactin (siderophore) exporter	-4.405109	4.07E-159
PMI39_04351	iron complex transport system substrate-binding protein	-3.797315	1.64E-77
PMI39_04362	Enamine deaminase RidA, house cleaning of reactive enamine intermediates, YjgF/YER057c/UK114 family	-3.60466	1.63E-58
PMI39_04363	cytosine deaminase	-4.406223	2.79E-33
PMI39_04364	vanillate O-demethylase ferredoxin subunit	-3.960255	6.06E-153
PMI39_04365	DNA-binding transcriptional regulator, LysR family	-3.875951	6.81E-147
PMI39_04644	LysR family transcriptional regulator, glycine cleavage system transcriptional activator	-3.834282	4.65E-62
PMI39_04645	2OG-Fe(II) oxygenase superfamily protein	-3.80261	1.55E-109
PMI39_04646	Protein of unknown function (DUF2817)	-3.855085	1.54E-109
PMI39_04647	lysine/arginine/ornithine transport system substrate-binding protein	-4.372496	4.31E-167
PMI39_04648	Pimeloyl-ACP methyl ester carboxylesterase	-4.877218	1.14E-55

PMI39_04649	two component heavy metal response transcriptional regulator, winged helix family	-3.840069	3.05E-151
PMI39_04650	two-component system, OmpR family, heavy metal sensor histidine kinase CusS	-4.256511	1.39E-192
PMI39_04651	nitroreductase / dihydropteridine reductase	-3.968208	1.25E-155
PMI39_04652	zinc-binding alcohol dehydrogenase family protein	-3.632172	1.91E-111
PMI39_04653	DNA-binding transcriptional regulator, LysR family	-3.338261	1.48E-67
PMI39_04654	transcriptional regulator, TetR family	-3.787312	7.94E-116
PMI39_04655	aspartate aminotransferase	-3.727061	1.98E-126
PMI39_04656	NADP-dependent 3-hydroxy acid dehydrogenase YdfG	-3.841054	2.92E-73
PMI39_04658	N-methylhydantoinase A	-3.441174	1.48E-111
PMI39_04659	MFS transporter, MHS family, proline/betaine transporter	-3.832215	1.90E-102
PMI39_04660	Regulator of RNase E activity RraA	-3.858822	1.21E-50
PMI39_04661	GntR family transcriptional regulator, uxuAB operon transcriptional repressor	-3.797028	2.78E-84
PMI39_04662	PTS system, cellobiose-specific IIB component	-4.115381	8.35E-48
PMI39_04663	6-phospho-beta-glucosidase	-4.737428	1.05E-83
PMI39_04664	PTS system, cellobiose-specific IIC component	-4.707848	3.44E-165
PMI39_04665	PTS system, cellobiose-specific IIA component	-4.033436	6.99E-55
PMI39_04666	Uncharacterized conserved protein, DUF1778 family	-4.553939	0
PMI39_04667	Acetyltransferase (GNAT) domain-containing protein	-4.742295	0
PMI39_04668	Threonine/homoserine/homoserine lactone efflux protein	-4.599564	0
PMI39_04688	2-dehydropantoate 2-reductase	-4.162174	8.40E-185
PMI39_04689	transcriptional regulator, DeoR family	-4.292522	1.01E-93
PMI39_04690	hypothetical protein	-4.250666	2.79E-56
PMI39_04691	NAD(P)-dependent dehydrogenase, short-chain alcohol dehydrogenase family	-4.206272	1.68E-84
PMI39_04692	DNA-binding transcriptional regulator, LysR family	-4.292098	6.65E-189
PMI39_04693	NAD(P)-dependent dehydrogenase, short-chain alcohol dehydrogenase family	-4.070633	1.15E-96
PMI39_04694	hypothetical protein	-3.897826	2.83E-61
PMI39_04695	iron complex transport system substrate-binding protein	-4.200988	4.18E-58
PMI39_04696	iron complex outermembrane recepter protein	-4.394581	7.15E-82
PMI39_04697	transcriptional regulator, RpiR family	-4.14306	7.52E-74
PMI39_04698	Threonine/homoserine efflux transporter RhtA	-4.272625	2.91E-108
PMI39_04699	Pimeloyl-ACP methyl ester carboxylesterase	-4.730906	1.27E-61
PMI39_04700	outer membrane autotransporter barrel domain-containing protein	-4.533139	8.00E-291
PMI39_04701	outer membrane receptor for ferrienterochelin and colicins	-4.32697	1.41E-228
PMI39_04702	polar amino acid transport system substrate-binding protein	-4.042142	8.59E-54
PMI39_04703	polar amino acid transport system permease protein	-4.064309	1.45E-76

PMI39_04704	polar amino acid transport system ATP-binding protein	-4.074336	6.98E-115
PMI39_04705	biotin/methionine sulfoxide reductase	-3.186627	3.66E-185
PMI39_04706	antitoxin ChpS	-3.767306	5.86E-75
PMI39_04904	L-alanine-DL-glutamate epimerase and related enzymes of enolase superfamily	-3.857193	2.24E-75
PMI39_04905	L-fuconolactonase	-3.759893	2.17E-89
PMI39_04906	L-fucose dehydrogenase	-3.673689	1.07E-70
PMI39_04907	L-rhamnose mutarotase	-4.271434	2.27E-82
PMI39_04908	MFS transporter, FHS family, L-fucose permease	-4.372326	2.95E-171
PMI39_04909	Putative motility protein	-4.229838	1.94E-90
PMI39_04910	2-desacetyl-2-hydroxyethyl bacteriochlorophyllide A dehydrogenase	-4.414943	7.56E-257
PMI39_04911	DNA-binding transcriptional regulator, LysR family	-4.359616	7.30E-132
PMI39_04912	Thymidylate kinase	-4.682217	3.47E-177
PMI39_04913	cold shock protein (beta-ribbon, CspA family)	-3.786584	2.96E-81
PMI39_04914	Peptidoglycan/LPS O-acetylase OafA/YrhL, contains acyltransferase and SGNH-hydrolase domains	-4.851216	8.09E-123
PMI39_04915	palmitoyl transferase	-4.131974	7.87E-91
PMI39_04916	Lysophospholipase, alpha-beta hydrolase superfamily	-3.907366	4.94E-171
PMI39_04917	Surface polysaccharide O-acyltransferase, integral membrane enzyme	-4.170569	1.83E-182
PMI39_04918	Multidrug efflux pump subunit AcrB	-4.208872	1.75E-215
PMI39_04919	RND family efflux transporter, MFP subunit	-3.874445	2.55E-51
PMI39_04921	potassium and/or sodium efflux P-type ATPase	-4.238932	4.04E-136
PMI39_04922	AI-2 transport protein TqsA	-4.337715	2.10E-159
PMI39_04923	Multidrug efflux pump subunit AcrB	-3.76538	3.32E-125
PMI39_04924	RND family efflux transporter, MFP subunit	-4.352664	3.69E-104
PMI39_04926	ATPase, P-type (transporting), HAD superfamily, subfamily IC	-3.877956	4.46E-120
PMI39_04927	protein of unknown function (DUF903)	-3.72936	1.19E-51
PMI39_04928	protein of unknown function (DUF903)	-3.721387	9.82E-50
PMI39_04929	GntR family transcriptional regulator	-3.962393	6.14E-70
PMI39_04930	succinyl-diaminopimelate desuccinylase	-4.017724	4.54E-117
PMI39_04931	peptide/nickel transport system substrate-binding protein	-3.837052	1.02E-178
PMI39_04932	peptide/nickel transport system permease protein	-3.807919	3.74E-41
PMI39_04934	peptide/nickel transport system ATP-binding protein	-3.803233	3.05E-154
PMI39_04935	acetylornithine deacetylase	-5.538087	1.59E-06
PMI39_04936	Xaa-Pro dipeptidase	-3.957725	4.61E-151
PMI39_04937	D-alanyl-D-alanine dipeptidase	-3.892351	1.13E-145
PMI39_04938	transcriptional regulator, TraR/DksA family	-3.83024	1.49E-54

PMI39_04940	Coenzyme F390 synthetase	-3.628753	5.94E-145
PMI39_04941	Acyl-CoA reductase (LuxC)	-3.962486	5.05E-203
PMI39_04942	3-oxoacyl-[acyl-carrier protein] reductase	-3.58063	2.98E-144
PMI39_04944	type VI secretion system secreted protein Hcp	-3.887603	6.46E-151
PMI39_04945	O-acetyl-ADP-ribose deacetylase (regulator of RNase III), contains Macro domain	-4.079793	1.52E-204
PMI39_04946	maltooligosaccharide ABC transporter membrane protein	-3.925197	1.85E-164
PMI39_04947	maltose/maltodextrin transport system permease protein	-3.900935	1.21E-144
PMI39_04948	maltose/maltodextrin transport system substrate-binding protein	-4.161742	5.55E-148
PMI39_04949	multiple sugar transport system ATP-binding protein	-3.732575	1.02E-71
PMI39_04950	maltoporin	-4.096643	3.34E-101
PMI39_04951	maltose operon protein	-3.764856	5.74E-106
PMI39_04952	4-alpha-glucanotransferase	-4.172782	6.35E-141
PMI39_04953	starch phosphorylase	-4.137186	8.41E-136
PMI39_04954	LuxR family transcriptional regulator, maltose regulon positive regulatory protein	-4.265099	0
PMI39_04966	MFS transporter, DHA1 family, arabinose polymer transporter	-3.973773	1.70E-68
PMI39_04967	DNA-binding transcriptional regulator, LysR family	-3.515575	1.66E-46
PMI39_04968	hypothetical protein	-3.975284	7.43E-159
PMI39_04969	Sugar phosphate permease	-3.918522	3.06E-218
PMI39_04970	6,7-dimethyl-8-ribityllumazine synthase	-4.440229	3.68E-138
PMI39_04972	glycine oxidase	-3.767547	4.93E-51
PMI39_04973	sulfur carrier protein	-3.96307	2.91E-24
PMI39_04974	thiazole synthase	-3.493134	1.34E-66
PMI39_04975	Molybdopterin or thiamine biosynthesis adenylyltransferase	-3.701978	8.41E-91
PMI39_04976	putative hydroxymethylpyrimidine transport system ATP-binding protein	-3.493996	2.70E-121
PMI39_04977	putative hydroxymethylpyrimidine transport system permease protein	-3.799816	2.48E-96
PMI39_04978	putative hydroxymethylpyrimidine transport system substrate-binding protein	-3.935247	2.44E-150
PMI39_04989	NitT/TauT family transport system substrate-binding protein	-3.842591	1.15E-86
PMI39_04990	NitT/TauT family transport system ATP-binding protein	-4.694812	3.66E-50
PMI39_04991	NitT/TauT family transport system permease protein	-3.751047	1.11E-72
PMI39_04994	filamentous hemagglutinin	-4.155844	1.11E-260
PMI39_04995	Hemolysin activation/secretion protein	-4.274873	2.30E-170
PMI39_04996	KDO II ethanolaminephosphotransferase	-4.583976	2.71E-251
PMI39_04997	Predicted transcriptional regulators	-4.397141	2.68E-100
PMI39_04999	Protein N-acetyltransferase, RimJ/RimL family	-4.183066	7.00E-95

PMI39_05000	non-ribosomal peptide synthase domain TIGR01720/amino acid adenylation domain-containing protein	-4.176868	4.37E-209
PMI39_05001	MbtH protein	-3.872643	8.54E-54
PMI39_05002	enterochelin esterase	-4.374362	6.44E-176
PMI39_05003	iron complex outermembrane recepter protein	-4.843568	0
PMI39_05004	hypothetical protein	-4.162545	7.93E-256
